# Cortistatin prevents glucocorticoid-associated osteonecrosis of the femoral head via the GHSR1a/Akt pathway

**DOI:** 10.1038/s42003-024-05795-5

**Published:** 2024-01-26

**Authors:** Yuan Gao, Yunhao You, Pengfei Zhang, Yang Yu, Zhaoning Xu, Hui Wei, Zhicheng Liu, Ruixuan Yu, Gaoxin Jin, Hao Wang, Shuai Zhang, Yuhua Li, Weiwei Li

**Affiliations:** 1 Department of Orthopedics, Qilu Hospital of Shandong University, Jinan, China; 2 Cheeloo College of Medicine, Shandong University, Jinan, China; 3 Department of Rehabilitation, Qilu Hospital of Shandong University, Jinan, China; 4 Department of Pathology, Qilu Hospital of Shandong University, Jinan, China; 5 Department of Trauma Orthopedics, Shandong Provincial Hospital Affiliated to Shandong First Medical University, Jinan, China

**Keywords:** Diseases, Bone, Apoptosis, Drug discovery

## Abstract

Long-term use of glucocorticoids (GCs) is known to be a predominant cause of osteonecrosis of the femoral head (ONFH). Moreover, GCs can mediate apoptosis of various cell types by exaggerating oxidative stress. We have previously found that Cortistatin (CST) antagonizes oxidative stress and improves cell apoptosis in several conditions. In this study, we detected that the CST expression levels were diminished in patients with ONFH compared with femoral neck fracture (FNF). In addition, a GC-induced rat ONFH model was established, which impaired bone quality in the femoral head. Then, administration of CST attenuated these ONFH phenotypes. Furthermore, osteoblast and endothelial cells were cultured and stimulated with dexamethasone (Dex) in the presence or absence of recombinant CST. As a result, Dex induced impaired anabolic metabolism of osteoblasts and suppressed tube formation in endothelial cells, while additional treatment with CST reversed this damage to the cells. Moreover, blocking GHSR1a, a well-accepted receptor of CST, or blocking the AKT signaling pathway largely abolished the protective function of CST in Dex-induced disorder of the cells. Taken together, we indicate that CST has the capability to prevent GC-induced apoptosis and metabolic disorder of osteoblasts in the pathogenesis of ONFH via the GHSR1a/AKT signaling pathway.

## Abstract (web summary)

Cortistatin (CST) may have the capability to prevent GC-induced apoptosis and metabolic disorder of osteoblasts in the pathogenesis of ONFH via the GHSR1a/AKT signaling pathway.

## Introduction

Osteonecrosis is a common clinically observed disease. Currently, increasing attention is being paid to attenuating this process, while much is still required for efficient therapy^1^. Osteonecrosis of the femoral head (ONFH) is defined as the cellular death of bone, which usually leads to the destruction of the hip joint and requires arthroplasty surgeries^2^. Steroid-induced ONFH is a severe side effect of glucocorticoid (GC) use, and excessive use of GCs in the clinic is a predominant cause of ONFH^3^. To date, the underlying mechanism of GC-induced ONFH still remains to be elucidated, but it appears that cell death, vascular compromise, or deficient bone repair are involved in this process^4^. High-dose GC treatment exaggerates apoptosis, and GC-induced apoptosis is considered to be a result of direct hormonal effects on bone cells^3^. GC-mediated apoptosis of bone cells contributes to the loss of bone strength as well as the loss of bone mineral density. Recent findings suggest that enhanced apoptosis of bone cells is associated with oxidative stress, which is associated with mitochondrial ROS^5^.

It has been reported that excess GC often causes damage to the vascular system. The endothelium is a critical site for the control of apoptosis during various processes, and the suppression of endothelial cell (EC) apoptosis is crucial for blood vessel integrity and angiogenesis^6^. GC treatment is known to inhibit the expression of VEGF-A and consequent vasculogenesis in ECs, which impairs the vascular system^7^. Previous reports showed that impairment of angiogenesis can induce ONFH, while enhancement of angiogenesis can prevent the progression of ONFH^8^.

Osteogenesis is severely affected by GC hormones. GCs have complex stimulatory and inhibitory effects on skeletal metabolism^9^. Exaggerated GC signaling activity is responsible for the apoptosis of osteoblasts and plays a detrimental role in osteogenesis^10^. Therefore, treatment with high-dose GCs diminishes bone mass and triggers a decline in bone quality, which in turn leads to bone loss^11^.

Cortistatin (CST) is a cyclic neuropeptide that displays multiple properties in several physiological as well as disease processes^12^. Recent findings in types of models have indicated that CST might represent a natural, endogenous protective factor against disorganization of apoptosis as well as metabolism^13,14^. Moreover, CST has exhibited potential as a therapeutic strategy for degenerative and inflammatory diseases^15,16^. Recently, we indicated that CST was protective in maintaining the normal structure and function of mitochondria and attenuated mitochondrial ROS in the degeneration of intervertebral discs^14^.

However, whether CST is involved in osteonecrosis is unknown. The objectives of this research were to elucidate the potential function and underlying mechanisms of CST in the ONFH process.

## Results

### CST expression level is diminished in GC-induced ONFH

**Fig. 1:**
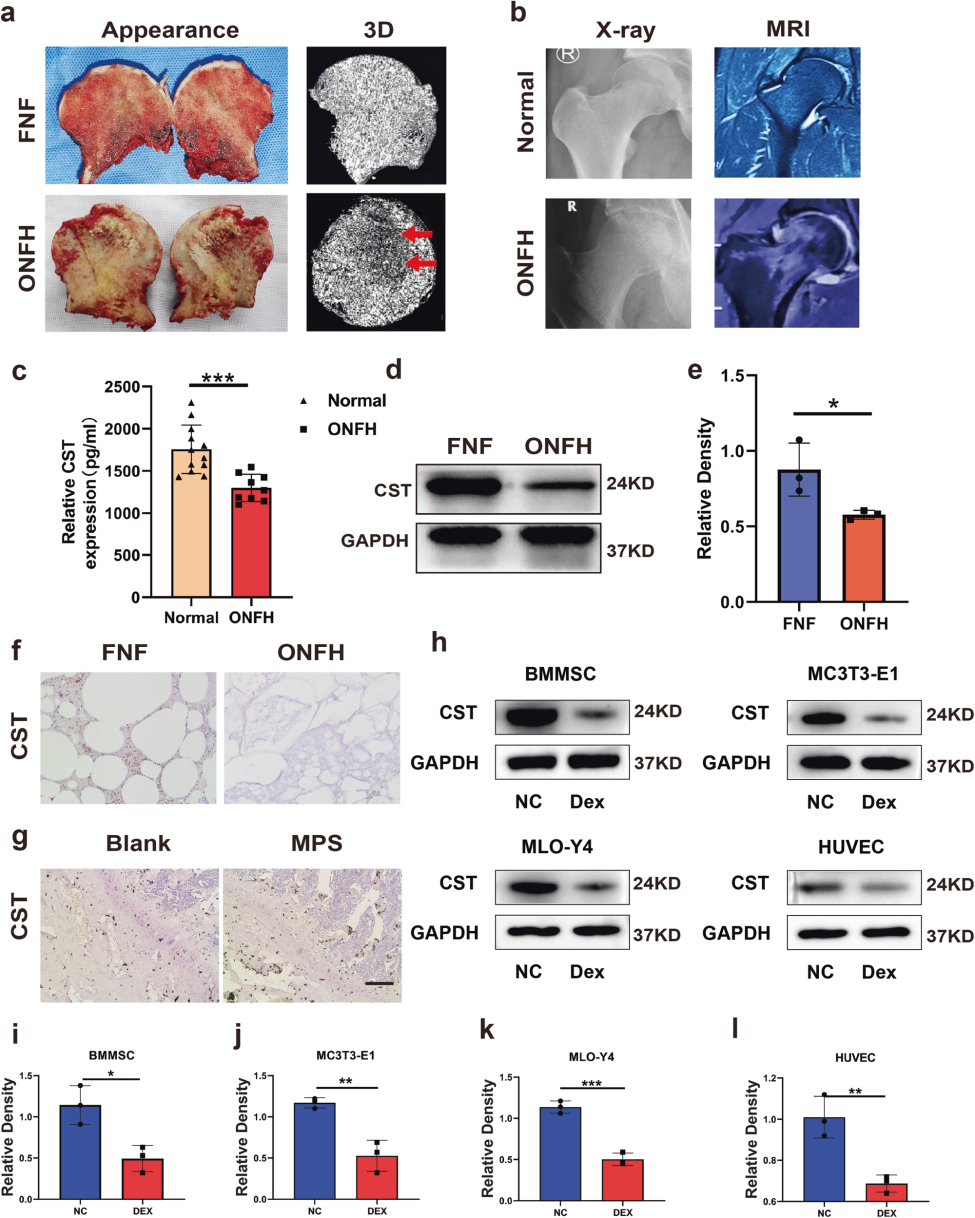
CST expression level is diminished in GC-induced ONFH. **a** The representative appearance and 3D reconstruction images of the femoral head of the FNF group (Control group) and ONFH group. The red arrow indicates the distinct collapse in weight-bearing area. **b** The representative X-ray and MRI images of the hip joint of normal patients and GC-induced ONFH patients. **c** The expression of serum CST level of normal and GC-induced ONFH patients. **d**, **e** Western blot (WB) analysis of CST expression of different groups of femoral-head tissue. **f** The representative immunohistochemistry pictures of CST expression in femoral-head tissue from the FNF group (Control group) and ONFH group. Scale bars, 100 μm. (**g**) The representative images of CST expression of MPS induced ONFH rat model. Scale bars, 100 μm. **h**–**l** The expression of CST in osteoblast stimulated by DEX. (*n* = 5 per group, data are shown as mean ± SD, ***p < 0.001, **p < 0.01, *p < 0.05).

In this study, the expression of CST was analyzed in GC-induced ONFH in the femoral-head tissues of both humans and rats. In comparison with the FNF group, the femoral head with steroid-induced femoral-head necrosis displayed a markedly abnormal gross appearance (Fig. 1a). For GC-induced ONFH, the section plane was in line with the X-ray and MRI images of the hip joint (Fig. 1b), compared with the FNF group. In addition, serum was collected from patients, and ELISA showed that the circulation level of CST was decreased in patients with ONFH (Fig. 1c). Western blot of protein extracts from the femoral-head tissue indicated that the expression of CST in GC-induced ONFH was reduced (Fig. 1d, e). Moreover, immunohistochemistry showed that CST expression was weakened in femoral-head tissue from patients with ONFH (Fig. 1f). In the rat model established by injection of lipopolysaccharide (LPS) and methylprednisolone (MPS), a similar trend of CST expression was also observed, and the ONFH model exhibited a reduced level of CST in the femoral head (Fig. 1g). To determine the role of GC in vitro, osteoblasts were cultured, and Western blot results indicated that Dex stimulation decreased the expression of CST (Fig. 1h–l).

### Supplementation with CST alleviates GC-induced ONFH

**Fig. 2:**
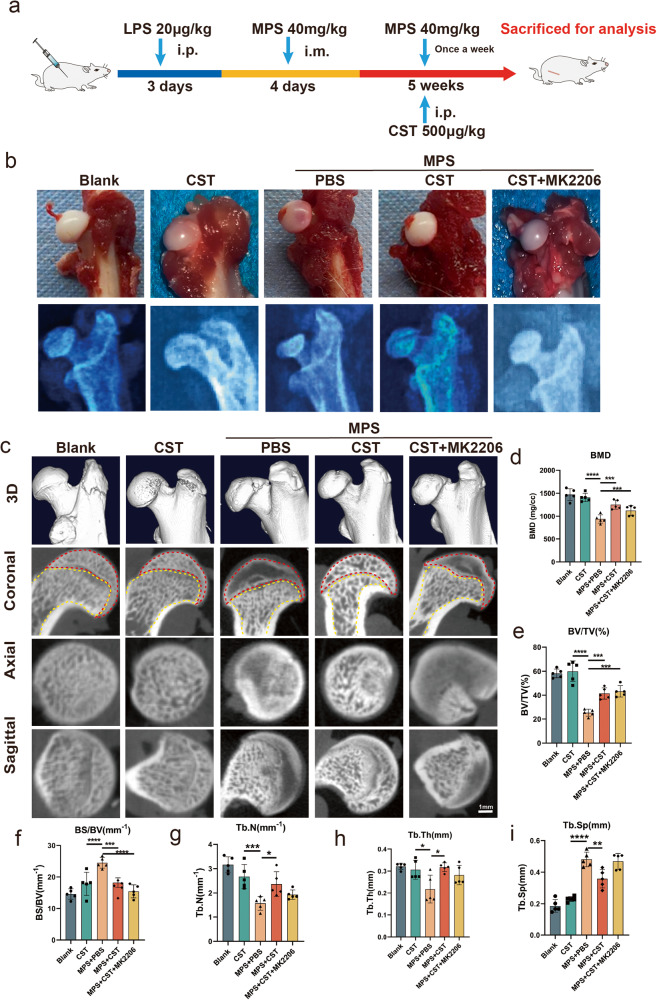
CST reduced necrosis of the femoral head in a rat model. **a** Schematic diagram illustrating the timeline of the operation process. The images and elements were created by authors. **b** The representative images and radiological manifestations of the femoral head in 1. The Blank group. 2. The CST group. 3. The MPS + PBS group (as a control group). 4. The MPS + CST group. 5. The MPS + CST + MK2206 group. **c** Micro-CT assay of 1. The Blank group. 2. The CST group. 3. The MPS + PBS group (as a control group). 4. The MPS + CST group. 5. The MPS + CST + MK2206 group. The red dashed area represents the subchondral bone, and the femoral neck is represented by a yellow dashed area. The statistical analysis of BMD (**d**), BV/TV (**e**), BS/BV (**f**), Tb.N (**g**), Tb.Th (**h**), and Tb.Sp (**i**) (*n* = 5 per group, data are shown as mean ± SD, ***p < 0.001, **p < 0.01, *p < 0.05).

**Fig. 3:**
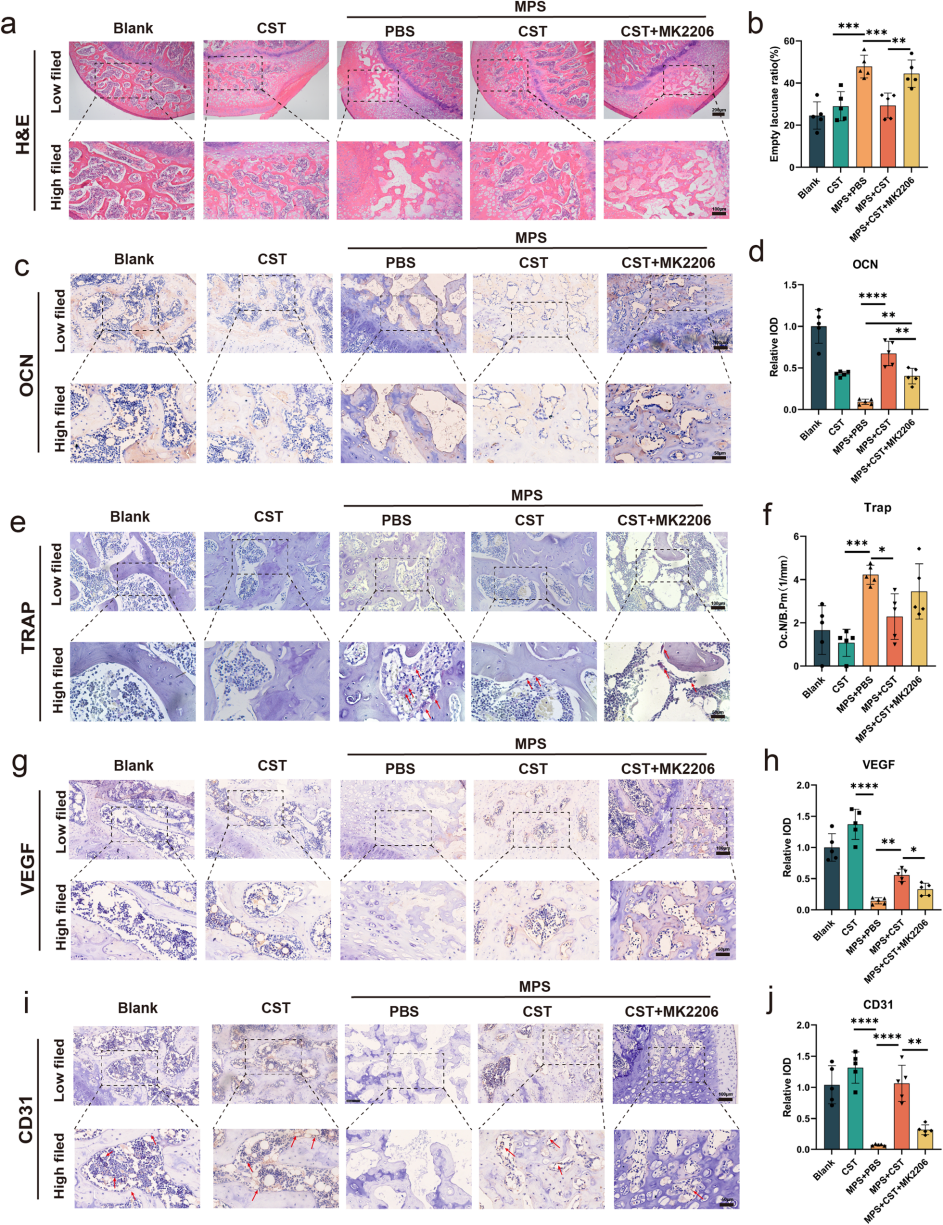
CST alleviated the progression of femoral-head necrosis in vivo. **a** Representative images of H&E stain of different indicated processing groups. Scale bars, 100 μm (low field), 50 μm (high field). **b** The statistical analysis of empty lacunae ratio of 1. The Blank group. 2. The CST group. 3. The MPS + PBS group (as a control group). 4. The MPS + CST group. 5. The MPS + CST + MK2206 group. **c**, **d** Immunohistochemical assay of osteocalcin in the femoral head of the indicated group. **e** The representative images of TRAP stain of different groups. The red arrow indicates Osteoclast. Scale bars, 100 μm (low field), 50 μm (high field). **f** The analysis of the number of osteoclasts. **g** Immunohistochemistry of VEGF in the femoral head of different groups. Scale bars, 100 μm (low field), 50 μm (high field). **h** Quantification of immunohistochemical analysis of the VEGF level of each group. **i**, **j** The representative images of CD31 stain of different indicated processing groups. The red arrow indicates blood vessels. Scale bars, 100 μm (low field), 50 μm (high field). (*n* = 5 per group, data are shown as mean ± SD, ***p < 0.001, **p < 0.01, *p < 0.05).

To investigate the function of CST, a GC-induced ONFH model was established in SD rats as described in the Methods, and recombinant CST peptide was administered through intraperitoneal injection (Fig. 2a). In the blank group, the cartilage surface of the femoral head was smooth and uniform in color, while the cartilage surface in the weight-bearing area of the model group was abnormal in color, and the surface was not smooth. Meanwhile, the use of CST alone has no significant effect on the femoral head (Fig. 2b). Nevertheless, treatment with exogenous CST reduced the severity of lesions (Fig. 2b), however, the use of MK2206 (AKT signaling pathway inhibitor) suppressed this phenomenon. In addition, CST treatment greatly relieved the radiological manifestations of GC-induced ONFH, as detected through X-ray (Fig. 2b). To further determine the morphological changes of the femoral head, micro-CT scans of the samples were performed, by which cortical as well as cancellous bone could be visually exhibited. From the sagittal, coronal, and cross-sectional views (Fig. 2c) and 3D reconstruction, it can be observed that the MPS + PBS group had severe trabecular bone loss and collapse in the weight-bearing area. CST treatment markedly alleviated the aforementioned femoral-head damage. However, the use of MK2206 suppresses this phenomenon (Fig. 2c). Based on micro-CT analysis of the region of interest (ROI) in the weight-bearing area, we detected that the mean BMD of the MPS group (982.0  ±  128.3 mg/cm3) was significantly lower than that of the normal group (1471  ±  116.6 mg/cm^3^). Intriguingly, we found that CST treatment improved BMD, BV/TV, Tb.N, and Tb.Th compared with the model group and reduced Tb.Sp and BS/BV (Fig. 2c–i).

The results of histological analysis further verified the attenuation of impaired bone formation via treatment with recombinant CST. In H&E staining, higher empty lacuna numbers and more trabecular collapse were observed in the MPS + PBS group, while CST treatment greatly reversed this trend (Fig. 3a, b). The use of inhibitor MK2206 inhibits the effect of CST (Fig. 3a, b). Meanwhile, we performed HE staining and photography (Scale bar:250μm) on the femoral neck to observe the bone trabeculae and found that there was no significant reduction in the bone trabeculae in the MPS group, which can be proved that there is no obvious evidence of osteoporosis (Supplementary Fig. 1). Bone histomorphometric analysis showed that CST administration maintained the osteoblast biomarker osteocalcin compared with that of the MPS + PBS group (Fig. 3c, d), which was consistent with the micro-CT findings. Tartrate-resistant acid phosphatase (TRAP) staining revealed that the osteoclast number was abnormally increased in the subchondral bone of the femoral head in the model group, and CST improved this phenomenon (Fig. 3e, f and Supplementary Fig. 2). Disturbed angiogenesis is a common morphological change in ONFH. In this study, immunostaining assays for VEGF (Fig. 3g, h and Supplementary Fig. 3) and immunohistochemistry for CD31 (Fig. 3i, j and Supplementary Fig. 4) were performed, and blood vessel formation was dramatically diminished by MPS, while the additional use of CST retained the angiogenic ability of the femoral head in the ONFH model.

### CST inhibited dysfunction of osteoblasts induced by Dex

**Fig. 4:**
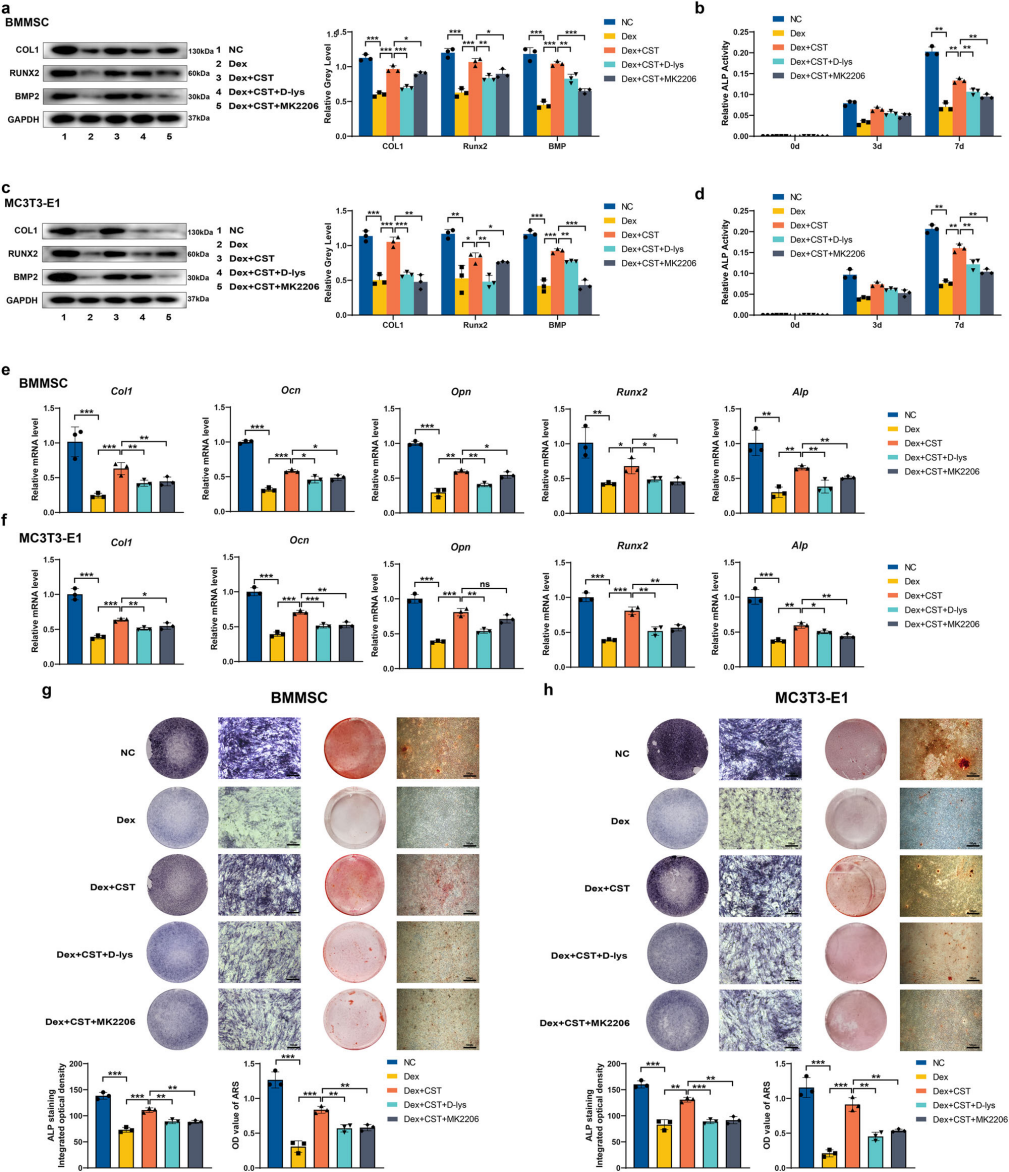
CST inhibited dysfunction of osteoblast induced by Dex in BMMSC and MC3T3-E1 cells. **a**, **c** Protein expression levels of COL1, Runx2, and BMP2 (day 5). **b**, **d** Relative ALP activity on day 0, day 3, day 7. **e**, **f** mRNA expression levels of COL1, OCN, OPN, Runx2, and ALP (day 3). **g**, **h** ALP staining (day 14) and ARS staining (day 14). Scale bar, 200 µm. (*n* = 3 per group, data are shown as mean ± SD, ***p < 0.001, **p < 0.01, *p < 0.05).

Damage to the osteogenic differentiation of BMMSC and osteoblast is one of the cytological causes of ONFH^17^. We wanted to investigate whether CST exerted a protective effect of Dex by protecting the osteogenic differentiation capacity. We used an osteoblastic differentiation-inducing medium to research the effects of CST on osteogenesis. Osteogenesis-related gene expression was assessed at the RNA and protein levels after drug treatment. The mRNA expression of ALP, COL1, OPN, OCN, and RUNX2 and the protein expression of COL1, Runx2, ALP, and BMP2 were downregulated after Dex treatment, which suggested that the osteogenic differentiation capacity was damaged by Dex. CST could rescue the expression of osteogenesis-related genes at both the RNA and protein levels. Therefore, BMMSC and MC3T3-E1 cells regained osteogenic ability with the assistance of CST (Fig. 4a, c, e, f). ALP staining, ALP activity assays, and Alizarin Red staining were performed to comprehensively evaluate osteogenic capacity (Fig. 4b, d, g, h). Dex distinctly lowered endogenous alkaline phosphatase activity and matrix mineralization, while CST significantly improved these indicators. D-Lys, [D-Lys3]-growth hormone-releasing peptide-6, is an antagonist of CST function receptor-growth hormone secretagogue receptor 1a (GHSR1a). MK2206 inhibits CST function in an Akt pathway-dependent manner. Both drugs can act as antagonists of CST (Fig. 4b, d, g, h). This demonstrated that D-lys or MK2206 could reverse the protective effect of CST from Dex on the osteogenesis of BMMSC and MC3T3-E1 cells to some degree, although OPN mRNA expression was not totally inhibited in BMMSC. We concluded that the effect of CST was partially mediated by the GHSR1a and AKT pathways.

### Protective effect of CST on cell apoptosis induced by Dex in vitro

**Fig. 5:**
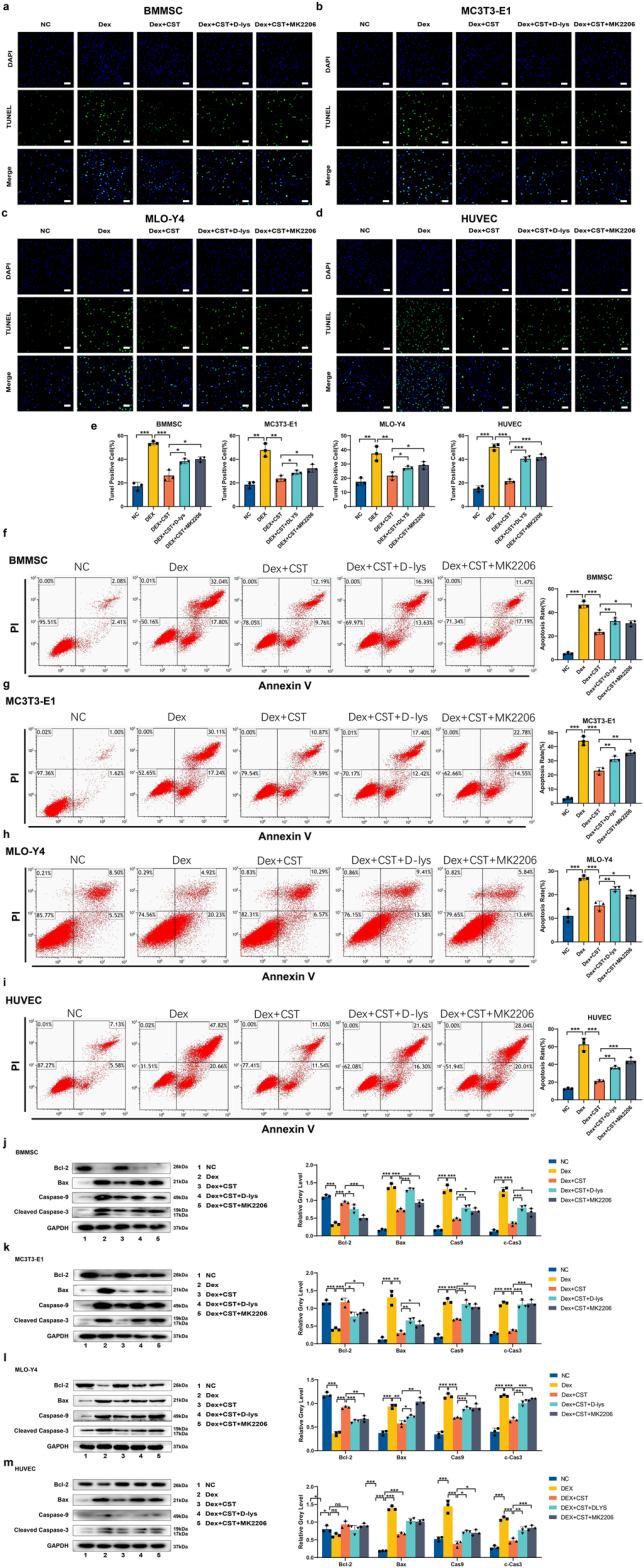
Protective effect of CST on cell apoptosis induced by Dex in vitro in BMMSC, MC3T3-E1, MLO-Y4, and HUVEC cells. **a**–**d** TUNEL staining of the BMMSC (**a**), MC3T3-E1 (**b**), MLO-Y4 (**c**), and HUVEC (**d**) cells of each indicated group. Nuclei were stained with DAPI. Scale bar, 100 µm. **e** Statistical graphs of Tunel staining. **f**–**i** Quantification of the BMMSC(f), MC3T3-E1 (**g**), MLO-Y4 (**h**), and HUVEC (**i**) cells cell apoptosis by flow cytometry. Protein expression levels of Bcl-2, Bax, Caspase-9, and Cleaved Caspase-3 of the BMMSC (**j**), MC3T3-E1 (**k**), MLO-Y4 (**l**) and HUVEC (**m**) cells. (*n* = 3 per group, data are shown as mean ± SD, ***p < 0.001, **p < 0.01, *p < 0.05).

It is known that GC-induced apoptosis is considered to be a reason for ONFH^18^. To study the treatment effect of CST, we used a TUNEL assay and flow cytometric analysis to assess the apoptosis rate of cells. These two results agree well with each other. Exposure to Dex dramatically increased the apoptosis rate compared to the control group. In contrast, supplementation with CST attenuated the apoptotic effect of Dex in BMMSC, MC3T3-E1, MLO-Y4, and HUVEC cells. It is known that CST binds to GHSR1a receptor^19^, together with the finding that CST interacts with the AKT signaling pathway which is critical in osteoblast function^20^, prompted us to investigate the potential involvement of these two CST-associated factors. As a result, supplementation with GHSR1a inhibitor D-lys or AKT signaling pathway inhibitor MK2206 partially reversed the protective effect of CST (Fig. 5a–i).

Then, we performed western blotting to show the expression levels of apoptosis-regulated proteins (caspase-9, cleaved caspase-3, Bax) and an inhibitor of apoptosis protein (Bcl-2) in BMMSC, MC3T3-E1, MLO-Y4, and HUVEC cells. After 48 h of exposure to Dex, caspase-9, cleaved caspase-3, and Bax were obviously elevated, and Bcl-2 was decreased, which was consistent with the apoptosis-promoting characteristic of Dex. However, CST addition effectively reversed the Dex-induced apoptosis effect at the molecular level. However, when treated with D-lys or MK2206, the expression of caspase-9, cleaved caspase-3, and Bax was elevated, and Bcl-2 was decreased to a certain extent (Fig. 5j–m).

### Effects of CST on mitochondrial dysfunction and oxidative stress induced by Dex

**Fig. 6:**
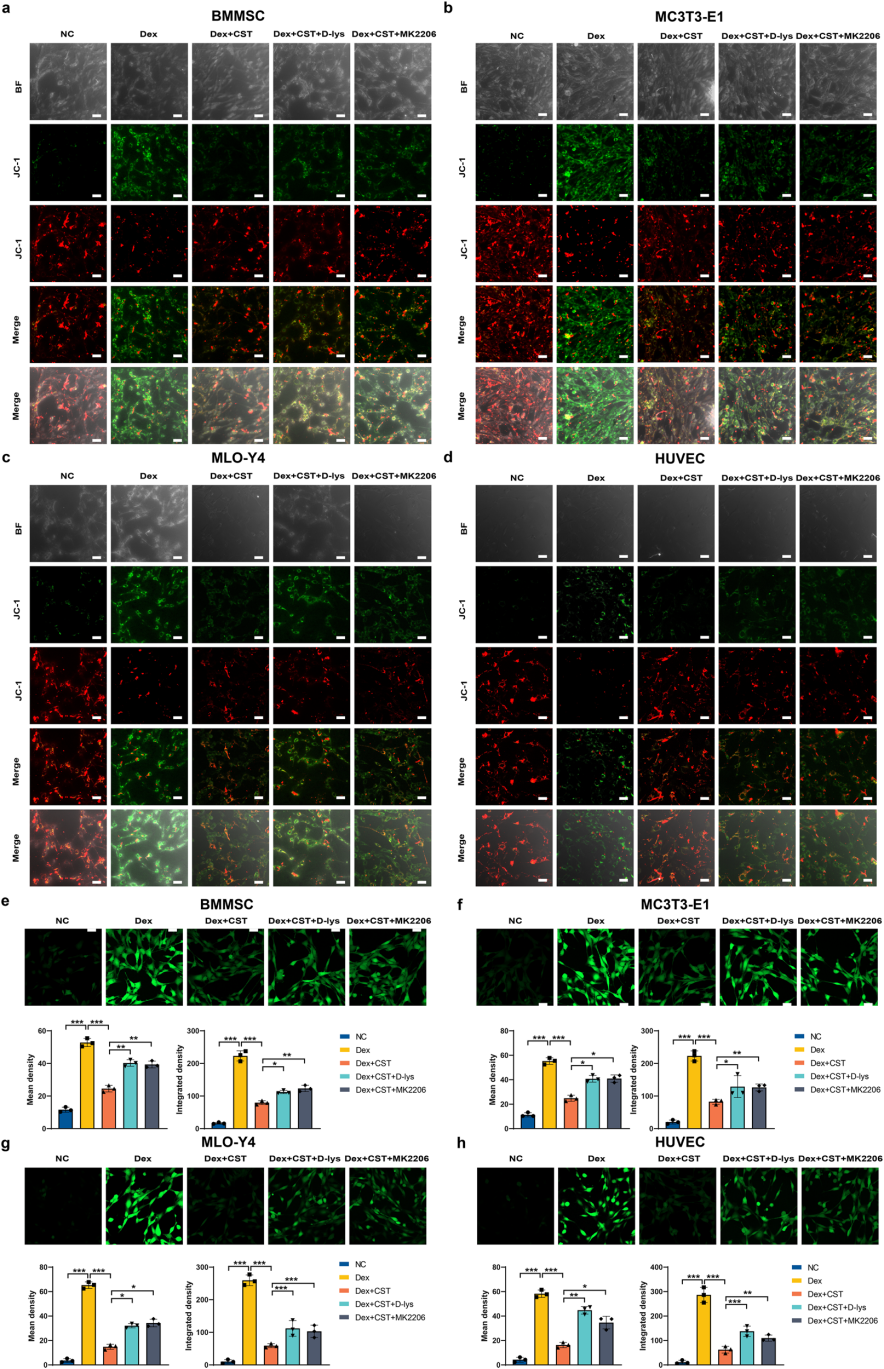
Effects of CST on mitochondrial dysfunction and oxidative stress induced by Dex in BMMSC, MC3T3-E1, MLO-Y4, and HUVEC cells. **a**–**d** JC-1 assay in each indicated group of the BMMSC (**a**), MC3T3-E1 (**b**), MLO-Y4 (**c**), and HUVEC (**d**) cells. **e**, **f** Representative images and quantification of ROS levels in each indicated group of the BMMSC (**e**), MC3T3-E1(**f**), MLO-Y4 (**g**), and HUVEC (**h**) cells. (*n* = 3 per group, data are shown as mean ± SD, ***p < 0.001, **p < 0.01, *p < 0.05. Scale bar, 100 µm).

The mitochondria in mammalian cells work as dynamic biophysical systems and play crucial roles in the regulation of energy metabolism to control cell death and survival^21^. The JC-1 assay is widely used in apoptosis studies to reflect mitochondrial function. JC-1 dye can enter intact mitochondrial membranes and aggregate to form fluorescent red, while it exists as monomers in the fractured mitochondrial membrane. In BMMSC, MC3T3-E1, MLO-Y4, and HUVEC cell lines, Dex enhanced FITC fluorescence intensity, and CST drastically reduced Dex-causing monomer aggregation. However, D-lys or MK2206 decreased the protective effect of CST (Fig. 6a–d). Since excess cellular levels of ROS lead to the activation of cell apoptosis, we performed a total reactive oxygen species assay to assess intracellular ROS production. The cells had strong fluorescence intensity after Dex treatment, which reflected excessive ROS production by Dex. ROS levels were obviously decreased in the Dex and CST groups. After D-lys or MK2206 was added, ROS levels were partially reversed compared with those in the Dex and CST groups (Fig. 6e–h).

### CST attenuates the impaired angiogenesis of HUVECs mediated by Dex

**Fig. 7:**
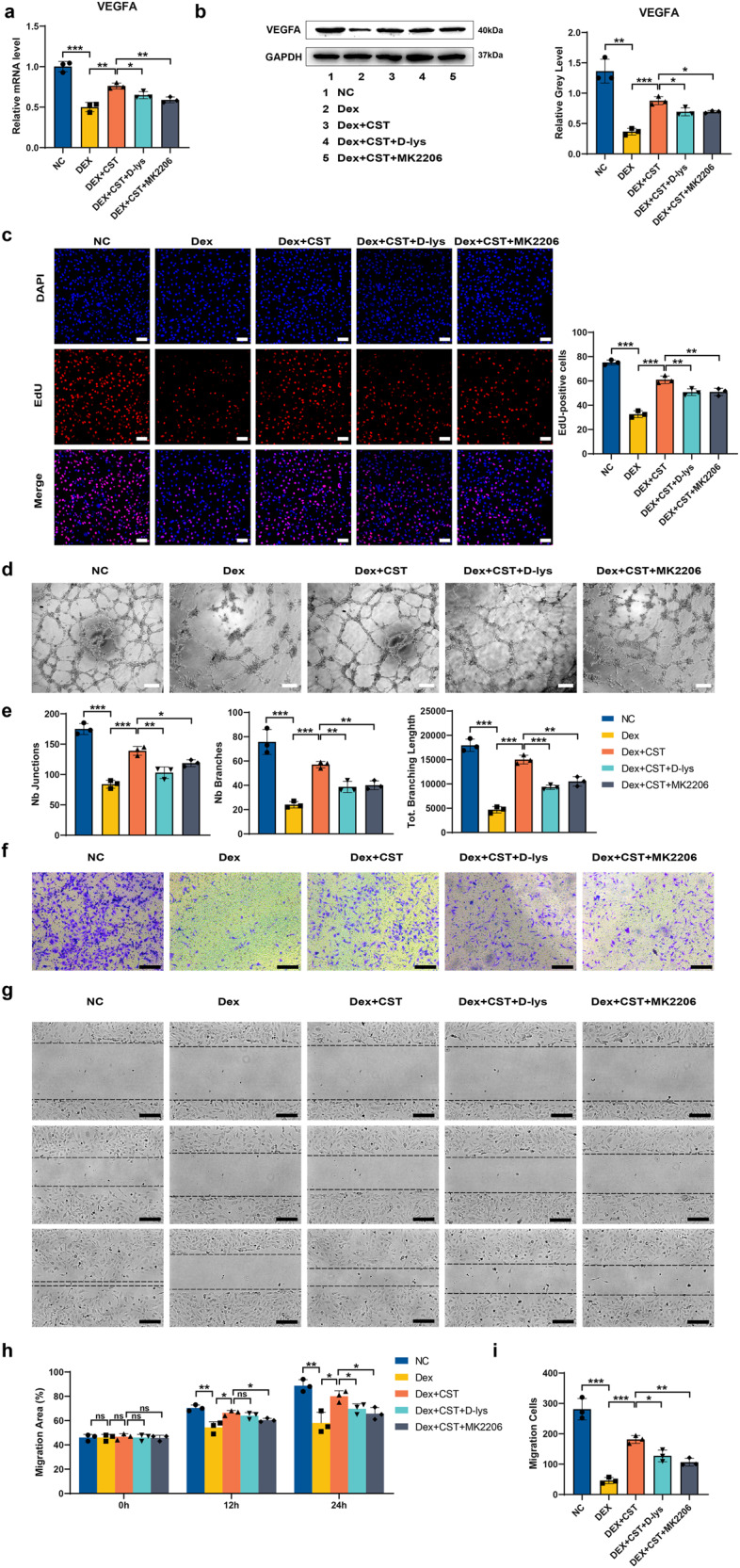
CST attenuates the impaired angiogenesis of HUVECs mediated by Dex. **a** mRNA expression levels of VEGFA under different concentrations for 48 h. **b** Protein expression levels of VEGFA. **c** EdU incorporation was visualized using a fluorescence microscope. The percentage of EdU-positive cells for each group was quantitated using ImageJ software (right graph). Scale bar, 100 µm. **d** Tube formation ability and quantitative analysis (**e**). Scale bar, 200 µm. **f** Transwell assay. **g** Scratched wound assay and quantitative analysis (**h**) for 0, 12, and 24 h. Scale bar, 200 µm. **i** Migration cells of Transwell assay. Scale bar, 200 µm. (*n* = 3 per group, data are shown as mean ± SD, ***p < 0.001, **p < 0.01, *p < 0.05).

Angiogenesis damage is an important pathogenic mechanism of femoral-head necrosis^22^, so we applied human umbilical vein endothelial cells (HUVECs) to assess endothelial cell function in vitro to further clarify the effect of CST on angiogenesis. The western blotting and PCR analysis results showed that the protein and mRNA expression levels of VEGFA were affected by Dex, but the use of CST showed that the expression was decreased by Dex. After D-lys or MK2206 was added, the protective effect of CST on the expression of VEGFA was partially diminished (Fig. 7a, b). We used the EdU assay and tube-formation assay to study HUVEC proliferation and angiogenesis. Dex inhibited the growth of HUVECs, as reported in the literature. However, CST treatment after 48 hours promoted HUVEC proliferation, which is consistent with the protective effect of CST on femoral-head necrosis. Meanwhile, supplementation with the two main antagonists, D-lys and MK2206, both inhibited the protective effect of CST on HUVEC proliferation (Fig. 7c). Junctions, branches, and branching length evaluation of the tube-formation assay quantitatively reflected the pro- or antiangiogenic potential of these substances. The addition of Dex markedly attenuated the tube-formation ability in all aspects, while CST reversed the Dex effect on tube formation. D-lys or MK2206 worked as CST antagonists and abolished the promoting effect of CST on tube formation (Fig. 7d, e). To study the migration ability of HUVECs, the scratch assay and the transwell assay were used. The scratch assay clearly showed that at 12 and 24 h, the healing ability of the Dex group was significantly slower than that of the normal group, while CST inhibited this effect of Dex (Fig. 7g, h). The transwell assay results showed that Dex seriously damaged the migration activity of HUVECs, and CST made up for this damage (Fig. 7f, i). Both the scratch and transwell assays showed that the migratory capacity of HUVECs was reduced by Dex and was restored after the application of CST, and the restoring effect of CST was partially attenuated after the later addition of D-lys or MK2206, which means that the protective effect of CST is partially mediated through the GHSR1a and AKT pathways.

## Discussion

GC-induced ONFH results from various factors, which can seriously affect bone reconstruction. As a challenging, disabling disease, ONFH has caused a global burden to patients and challenges to the medical community^23^. Based on a retrospective study that included 6395 ONFH patients in an Asian population, it was reported that 24.1% of all ONFHs were associated with GC treatment, in which autoimmune diseases were the preliminary cause of GC-induced ONFH^24^. Growing evidence reveals that GCs can lead to a reduction in bone density and are directly or indirectly associated with trabecular abnormalities as well as replacement with necrotic tissue, which lacks mechanical strength^25^. However, osteoporosis usually occurs in the cancellous bone in the intertrochanteric region of the femur and the femoral neck, rather than in the femoral head, and is associated with a lack of stress stimuli^26^. In the early-stage of ONFH, no obvious change can be detected in the histological appearance of the femoral head. However, due to mechanical stress overloading, the accumulation of bone metabolism disorders might eventually trigger the collapse of the femoral head^27^. The best opportunity for the treatment of GC-induced ONFH might be early-stage intervention, and therapeutic GC usage actually provides an available therapeutic window for the strategy of ONFH treatment. Therefore, investigating early-stage treatment instruments seems to carry greater clinical value.

To date, the LPS/MPS-induced rat model is considered to be an ideal preclinical model for mimicking GC-induced ONFH^28^. In the present study, we established typical end-stage ONFH pathological alterations, which is in line with previous studies^29^. Intriguingly, the administration of CST attenuated ONFH in the rat model, together with the protective role of CST in Dex-stimulated osteoblasts, HUVECs, and stem cells in the current study, implying that CST might be a potential therapeutic instrument for GC-induced ONFH. In the patients with ONFH, BMD, bone volume, and trabecular thickness were all markedly reduced in the collapsed zone, which is the load-bearing area, while abnormally thickened bone trabeculae were observed in the noncollapsed area^30^. These alterations are associated with GC-induced disorders of osteoblasts and osteoclasts, which would otherwise organize the homeostasis of bone formation and absorption^29^. In the present study, the results of micro-CT and histological analysis confirmed that the subchondral bone of the weight-bearing area in the ONFH rat model was protected by CST treatment, which was consistent with our findings in vitro. These data suggest that the effect of CST on maintaining bone quality in ONFH might be related to the regulation of osteoblasts and osteoclasts. GC-induced osteoblastogenesis disability can affect bone quality directly and has been investigated as a predominant target for ONFH therapy^31^. GCs can enhance cell apoptosis and inhibit the metabolism of osteoblasts and stem cells, which are commonly detected in ONFH. We recently discovered the potential of CST against titanium particle-induced osteolysis and osteoblast dysfunction^11^. Moreover, CST exhibits antioxidant function and can sustain the homeostasis of stem cells, which might provide its potential to improve stem cell function during ONFH^32^.

As a highly vascularized tissue, angiogenesis dramatically affects bone reconstruction^33^. Studies have proven that GCs display antiangiogenic properties by disturbing the migration ability of endothelial cells. Abnormal microthrombus formation is detected in the necrotic area of the femoral head. Impaired angiogenesis is the main reason for insufficiency of nutrients and oxygen, which would cause loss of viability for resident cells of the femoral head^34^. CST is expressed in blood vessels and plays a critical role in the homeostasis of blood vessel function^35^. In this study, the ability of HUVECs to migrate and form tubes was retained by CST treatment in the stimulation of high-dose GCs. Studies have shown that VEGF participates in osteogenesis–angiogenesis coupling^36^, and VEGF directly reflects angiogenesis and indirectly stimulates osteogenesis^37^. CD31, on the other hand, reflects the existence of blood vessels in the femoral head, which implies the severity of ONFH^38^. In vivo, the expression levels of VEGF and CD31 were reduced in GC-induced ONFH, while additional CST treatment compensated for this, which implies that CST attenuates devascularization of the femoral head mediated by GCs.

**Fig. 8:**
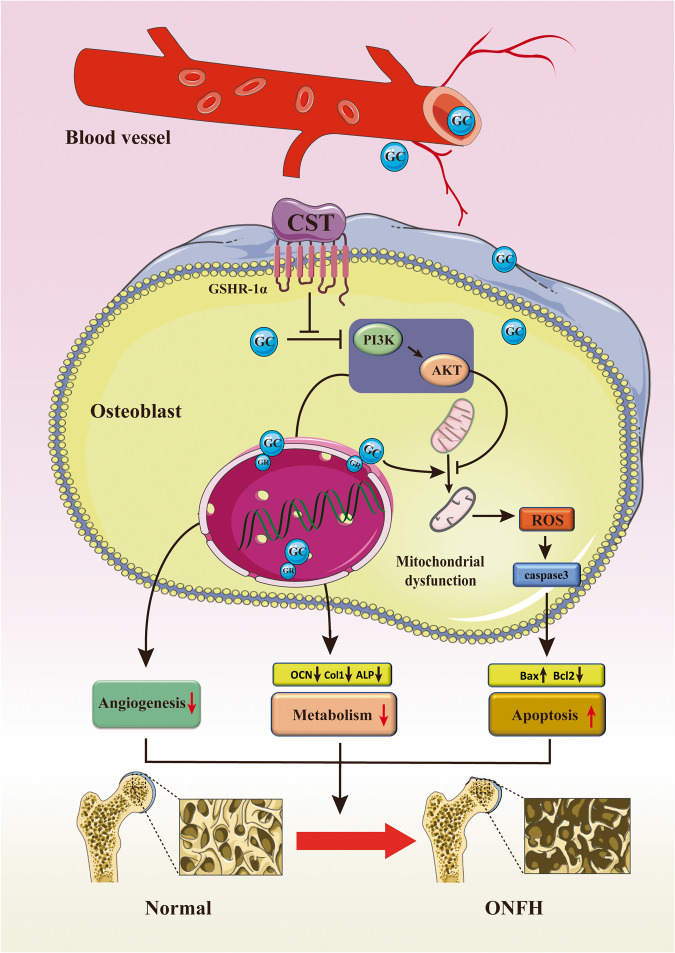
Schematic diagram of the protective effect of CST on osteoblasts. Schematic model of Cortistatin prevents disorganized apoptosis and metabolism of osteoblast in glucocorticoid-associated osteonecrosis of the femoral head via the GHSR1a/Akt pathway. The Figure was partly generated using Servier Medical Art, provided by Servier, licensed under a Creative Commons Attribution 3.0 Unported License.

One explanation for this is that CST binds to GHSR1a and retains the activity of the PI3k/AKT signaling pathway, which is suppressed by GCs. The AKT signaling pathway is well accepted in bone regeneration and plays a critical role in the homeostasis of bone structure during ONFH^39^. In the present study, GC usage repressed the activity of this signaling pathway, which further affected the osteogenesis of stem cells and interfered with the normal function of vascular epithelial cells, which is consistent with previous studies. Nevertheless, CST supplementation blocked the inactivation of the PI3k/AKT signaling pathway and alleviated ONFH development. Interestingly, blocking the PI3k/AKT pathway through MK2206 almost abolished the protective effect of CST, which suggests that CST might rely on the PI3k/AKT signaling pathway in regulating GC-induced disorganization (Fig. 8). GHSR1a is a receptor for CST, which is associated with osteogenesis and bone metabolism. In this study, the role of CST was inhibited by an antagonist of GHSR1a, D-lys, which might provide clues that the function of CST in ONFH might be facilitated through binding to GHSR1a.

## Methods

### Animals and grouping

All experimental procedures were completed under the Care and Use of Laboratory Animals guidance and approved by the Animal Ethics Committee of Qilu Hospital Shandong University. A total of 25 male Sprague–Dawley (SD) rats (10 weeks old, 300  ±  30 g) were obtained from Beijing Vital River Laboratory Animal Technology Co., Ltd. (Beijing, China) and were randomly divided into three groups. The sham group was used as a control, and GC-induced ONFH models were established in the other two groups: the PBS treatment control group and the CST treatment group.

### Model setup and drug treatment

Twenty-five Sprague–Dawley (SD) rats (male, age: 10 weeks, weight: 400 ± 50 g) were obtained from the Beijing Vital River Laboratory Animal Technology Co., Ltd. The rats were housed under controlled identical specific pathogen-free (SPF) standard environmental conditions (23 ± 2°C, 12 h light/dark cycle) with free access to food and allowed to move freely. The rats were divided into 5 groups (*n* = 5): (1) The Blank group. (2) The CST group which is only treated with CST. (3) The MPS + PBS group. (4) The MPS + CST group. (5) The MPS + CST + MK2206 group. GC-induced ONFH models were constructed as previously reported^40^. Briefly, the rats were weighed before the drug dose. Daily intraperitoneal (i.p.) injection of LPS (20 μg/kg, Sigma–Aldrich, USA) was administered for the first 3 days. Then, an intramuscular (i.m.) dose of methylprednisolone (MPS, 40 mg/kg, Pfizer, USA) was administered each day for the next 4 days and once a week for the next 5 weeks. MPS was injected into the left and right gluteus muscles alternately. The MPS + CST group was treated with recombinant CST (500 μg/kg, GL Biochem, China) twice a week via i.p. injection, and MPS + CST + MK2206 group was treated with recombinant CST twice a week via i.p. injection and MK2206 (360 mg/kg, ApexBio Technology, USA) via oral administration. The control group received PBS. Animals were sacrificed for femoral heads 5 weeks after MPS medication.

### Micro-CT analysis

To assess the imaging changes, the femoral heads of rats were scanned and analyzed using a high-resolution micro-CT Quantum GX2 (PerkinElmer, Japan). The scanning protocol included an isometric resolution of 15 μm, with X-ray energy settings of 70 kV and 200 μA. The ROI for quantitative analysis was the weight-bearing area of the femoral head under the articular cortical bone. Three-dimensional (3D) image reconstruction was created, and morphometric parameters were evaluated, including bone mineral density (BMD), bone volume to total volume fraction (BV/TV), bone surface/bone volume fraction (BS/BV), trabecular number (Tb.N), trabecular thickness (Tb.Th), and trabecular separation (Tb.Sp). To better assess the severity of femoral-head collapse, the area between the upper edge of the femoral head and the tangent circle was calculated in the coronal image. In addition, the cortical change of the femoral head was also recorded.

### Histological and immunohistochemical staining

Femoral-head samples were fixed for 48 h in 4% paraformaldehyde and decalcified for 4 weeks in 10% ethylenediaminetetraacetic acid (G1105, EDTA, Servicebio, China). Then, the samples were embedded in paraffin and cut into 6 μm thick slices. H&E staining was performed to observe the general view of specimens and to evaluate the trabecular structure. For TRAP staining, paraffin sections were stained with a TRAP staining kit (G1050, Servicebio, China). Briefly, the paraffin sections were stained with tartrate buffer containing naphthol AS-BI phosphate and pararosaniline chloride at 37 °C for 1 h in darkness and counterstained with hematoxylin. Section images were acquired using an IX71-SIF microscope (Olympus, Japan). IHC staining was performed to define the expression of CST, as well as osteogenesis-, vascular-, and pathway-related markers. In brief, sections were dewaxed and gradient hydrated to retrieve antigens. Then, primary antibodies, including CST, osteocalcin (ab133612), VEGFA (ab52917), and CD31 (ab9498), and corresponding secondary antibodies (all from Abcam Cambridge, UK) were incubated. The chromogenic reaction was induced by a DAB Kit (Beyotime, China). The tissue sections were observed with an IX71-SIF microscope (Olympus, Japan). IHC-positive cells and areas were measured using ImageJ and counted by two independent observers.

### TUNEL assay

TdT-mediated dUTP nick-end labeling (TUNEL) assays were performed with a one-step TUNEL apoptosis assay kit (C1088, Beyotime Institute of Biotechnology) according to the manufacturer’s instructions.

### Human tissue

Human femoral heads and blood were obtained from total hip arthroplasties at Qilu Hospital Shandong University, with at least five samples in each group. Briefly, the femoral head that was cut during the surgery was first cleaned with physiological saline and stored on ice, followed by Micro-CT examination. Subsequently, we selected the site of femoral-head collapse to extract bone tissue and perform pathological and western blotting assay. The blood obtained from the patient’s body is centrifuged at 3000 rpm for 20 min to obtain plasma, which is used for ELISA. Patients involved in the study provided consent, and the study was approved by the medical ethics regulations of the Medical Ethical Committee of Qilu Hospital of Shandong University (KYLI-2021(KS)−622). The femoral neck fracture samples were used as the control group, and the samples from patients diagnosed with GC-induced ONFH were used as the observation group.

### Cell extraction and methods

Murine bone marrow mesenchymal stem cells (BMMSC), mouse embryo osteoblast precursor cells (MC3T3-E1), MLO-Y4, and human microvascular endothelial cells (HUVEC) were purchased from Zhong Qiao Xin Zhou Biotechnology Co., Ltd. (Shanghai, China). BMMSC were cultured in DMEM/F12 (1:1) medium (HyClone, Logan, Utah, USA) containing 10% fetal bovine serum (FBS) and 1% double antibiotics (penicillin/streptomycin mix) (Gibco, Rockville, MD, USA). MC3T3-E1 cells were maintained in α-MEM (HyClone; GE Healthcare, Little Chalfont, UK) with 10% fetal bovine serum (10099141 C, FBS, Gibco), 100 U/mL penicillin, and 100 μg/mL streptomycin. HUVECs were cultured in MCDB131 medium (Gibco) supplemented with 5% FBS (Gibco), 2 mM L-glutamine (Gibco), 10 ng/mL epidermal growth factor (EGF, Sigma–Aldrich, St. Louis, MO, USA) and 1 μg/mL hydrocortisone (Sigma–Aldrich). The concentration of Dex used was 100 μM in the viability experiment, proliferation experiment, and osteogenic differentiation experiment, including analysis by Alizarin Red staining, western blotting, and PCR; 600 μM in the apoptosis experiment, including analysis by flow cytometry, western blotting, TUNEL assay; and 10 μM in the HUVEC experiment.

### Flow cytometry

Briefly, cultured BMMSC, MC3T3-E1, and MLO-Y4 cells from each indicated group were detected by flow cytometry. Cells were stained with propidium iodide (PI) and Annexin V-FITC for 15 min at room temperature in the dark in accordance with the protocol of the BD Pharmingen FITC Annexin V Apoptosis Detection Kit I (556547, BD Biosciences, USA)^41^. Cell apoptosis was assayed with a CytoFLEX S flow cytometer (Beckman Coulter, USA). The Gating Strategy was provided in Supplementary Fig. 6 in Supplementary Information. The data obtained from this assay were analyzed with FlowJo software.

### Real-time quantitative PCR

Total mRNA was isolated from BMMSC, MC3T3-E1, and HUVEC cells in each group using TRIzol Reagent (Beyotime Biotechnology Corporation, Shanghai, P.R. China). Total mRNA was used to synthesize the complementary DNA with the PrimeScript RT Reagent Kit (Vazyme Corporation, Nanjing, P.R. China). Real-time PCR was performed using an RNA PCR kit (Vazyme Corporation, Nanjing, P.R. China). All procedures were performed according to the manufacturer’s protocols. The PCR primer sequences used in the study are listed in Table 1.

**Table 1:** Sequence of primers used for real-time PCR.

Genes	Sense	Antisense
*GAPDH*	AAATCCCATCACCATCTTCCAG	AGGGGCCATCCACAGTCTTCT
*COL1*	CCCTGGTCCCTCTGGAAATG	GGACCTTTGCCCCCTTCTTT
*RUNX2*	GGGACTGTGGTTACCGTCAT	ATAACAGCGGAGGCATTTCG
*ALP*	GCACCTGCCTTACCAACTCT	GTGGAGACGCCCATACCATC
*OCN*	TCTGACCTCACAGATGCCAAG	AGGGTTAAGCTCACACTGCT
*OPN*	CACATGAAGAGCGGTGAGTCT	CCCTTTCCGTTGTTGTCCTG
*VEGFA*	ATAAGTCCTGGAGCGTTCCCT	GGTGAGAGATCTGGTTCCCG

### Western blot analysis

Proteins in BMSCs were obtained by lysing cells in a radioimmunoprecipitation assay (RIPA; Beyotime). A bicinchoninic acid kit for protein (BCA kit; Sigma–Aldrich) was used to quantify the protein concentration. After protein was separated and transmitted to a polyvinylidene fluoride membrane (Bio–Rad Laboratories), the membrane was blocked (Beyotime) and incubated with primary antibodies overnight at 4 °C, including anti-VEGFA (1:1000; ab52917; Abcam, USA), anti-caspase-9 (1:1000; 9508 T; Cell Signaling Technology, USA), anti-cleaved-caspase-3 (1:1000; 9661 T; Cell Signaling Technology, USA), anti-Bcl-2 (1:1000; ab182858; Abcam, USA), anti-Bax (1:1000; ab32503; Abcam, USA), anti-COL1 (1:1000; ab260043; Abcam, USA), anti-BMP (1:1000; ab214821; Abcam, USA), anti-Runx2 (1:1000; 12556S; Cell Signaling Technology, USA), and anti-GAPDH (1:1000; 5174 T; Cell Signaling Technology, USA). After incubation with the corresponding secondary antibody, the protein bands were captured by enhanced chemiluminescence (ECL; Sigma–Aldrich). The relative gray level was measured for quantitative analysis using Image Lab 3.0. The Original Western Blot images (Supplementary Fig. 5) have been provided in the Supplementary Information.

### Alkaline phosphatase and Alizarin Red staining

Alkaline phosphatase (ALP) staining was performed for culture in an osteogenic medium for 14 days, and BCIP/NBT (C3206, Beyotime) working solution was used for incubation. Alizarin Red staining (ARS) was performed for culture in an osteogenic medium for 21 days using an ARS staining solution (G1038, Servicebio, China). Pictures of well plates and images captured by microscopy were preserved on a quantitative basis. To quantify matrix mineralization, the alizarin red S-stained cultures were incubated with 100 mM cetylpyridinium chloride for 1 h to solubilize and release calcium-bound alizarin red into solution. The absorbance of the released alizarin red S was measured at 570 nm using a spectrophotometer.

### Transwell migration assay

HUVECs were preconditioned and plated into the upper chambers of a transwell plate (Corning). A complete culture medium was used as a chemoattractant and placed in the lower chamber. Then, 24 h later, the membranes were fixed and stained with crystal violet (Beyotime), and the membranes were mounted and observed with a light microscope.

### Scratch assay

HUVECs were cultured in 6-well plates to a confluent monolayer. Two separate wounds were scratched using a pipet tip, and the cells were rinsed with serum-free medium. Pictures at the same position of the wound were taken by microscopy at 0, 12, and 24 h. Migration ability was analyzed by quantifying the wound-healing area using Image-Pro Plus software (IPP, Media Cybernetics, Rockville, MD, USA).

### Tube formation

Tube formation assays were performed to evaluate the capillary-like structure formation of HUVECs. HUVECs were washed twice with serum-free medium, suspended in serum-free medium, and plated on angiogenesis plates. The extent of tube formation was assessed 6 h after seeding. IPP software was used to quantify tube length and branch points.

### JC-1 assay

A JC-1 assay kit (C2006; Beyotime Biotechnology) was used to detect the mitochondrial membrane potential in this study. After experimental operations, BMMSC and MC3T3-E1 cells in 24-well plates were stained with a JC-1 staining solution at 37 °C for 20 minutes while protected from light. Then, each well in the plate was washed twice with 1× JC-1 staining buffer, and the fluorescence intensity was measured with an LSM780 laser scanning confocal microscope (ZEISS, Germany). The red-to-green fluorescence ratio reflected changes in the mitochondrial membrane potential.

### Reactive oxygen species assay

An ROS assay kit (S0033; Beyotime Biotechnology) was used to detect intracellular reactive oxygen species (ROS). All procedures were performed in accordance with the manufacturer’s instructions. Briefly, BMMSC and MC3T3-E1 cells were stained with 10 μM DCFDA at 37 °C for 20 minutes in the dark and mixed every 4 minutes. Then, we washed the cells with serum-free culture medium three times. The DCFDA fluorescence intensity in each group was measured with an LSM780 laser scanning confocal microscope (ZEISS, Germany).

### Statistics and reproducibility

All experiments were repeated twice with at least three replications. The results are expressed as the mean ± standard deviation (SD), and GraphPad Prism 7.0 (GraphPad Software, Inc., USA) was used for the analysis. One-way ANOVA was used to determine statistical significance. Differences were considered significant at *p*  <  0.05.

### Reporting summary

Further information on research design is available in the Nature Portfolio Reporting Summary linked to this article.

### Supplementary information

Supplementary Information

Description of Additional Supplementary Files

Supplementary Data

Reporting Summary

## Data Availability

The source data for the graphs has been provided with the manuscript as Supplementary Data, and the Original Western Blot images (Supplementary Fig. 5) and The Gating Strategy (Supplementary Fig. 6) have been provided in the Supplementary Information file. All other data are available from the corresponding author.
