# Supplementary material for: Cortistatin prevents glucocorticoid-associated osteonecrosis of the femoral head via the GHSR1a/Akt pathway
Source: Commun Biol. 2024 Jan 26;7:132. doi: 10.1038/s42003-024-05795-5 (PMC10817896; doi:10.1038/s42003-024-05795-5)

Cortistatin prevents glucocorticoid-associated osteonecrosis of the femoral head via the GHSR1a/Akt pathway

Yuan Gao[1, *], Yunhao You[1,2, *], Pengfei Zhang[1, 2, *], Yang Yu[1, 2, *] Zhaoning Xu[2], Hui Wei[3], Zhicheng Liu[2,4], Ruixuan Yu[1, 2], Gaoxin Jin[2,4], Hao Wang[5, ¶], Shuai Zhang[1, ¶], Yuhua Li[1, ¶], Weiwei Li[4, ¶]

1. Department of Orthopedics, Qilu Hospital of Shandong University, Jinan, China
2. Cheeloo College of Medicine, Shandong University, Jinan, China
3. Department of Rehabilitation, Qilu Hospital of Shandong University, Jinan, China
4. Department of Pathology, Qilu Hospital of Shandong University, Jinan, China
5.Department of Trauma Orthopaedics, Shandong Provincial Hospital Affiliated to Shandong First Medical University, Jinan, China.

* These authors contribute equally.

¶To whom correspondence should be addressed: Hao Wang, Email: wanghaosl@sdfmu.edu.cn.; Shuai Zhang, Email: zhangshuai1106@126.com; Yuhua Li, Email: liyuhua@qiluhospital.com.; Weiwei Li, Email: liweiweizhao@163.com.

This file includes:

Supplementary Figure 1-6

Supplementary Figure 1

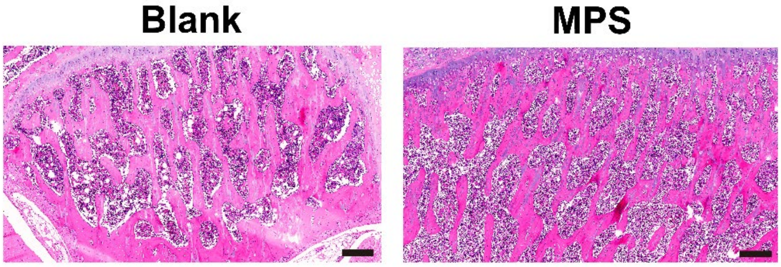

Supplementary Figure 1. Representative HE staining images of the femoral neck of rats in Blank and MPS groups (Scale bar:250μm)

Supplementary Figure 2

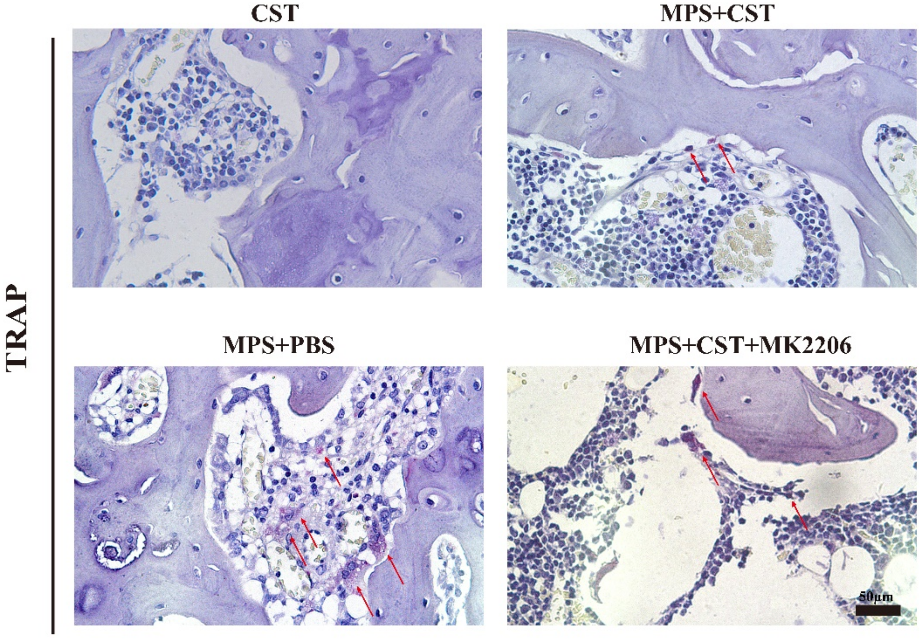

Supplementary Figure 2. High-definition images of (1) The CST group. (2) The MPS+PBS group (as control group). (3) The MPS+CST group. (4) The MPS+CST+MK2206 group in Figure 3E (Scale bar:50μm).

Supplementary Figure 3

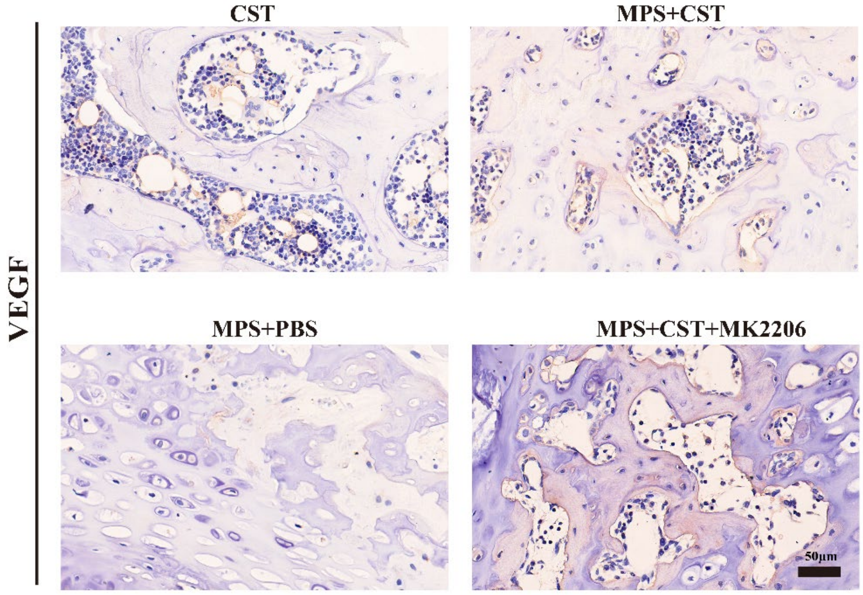

Supplementary Figure 3. High-definition images of (1) The CST group. (2) The MPS+PBS group (as control group). (3) The MPS+CST group. (4) The MPS+CST+MK2206 group in Figure 3G (Scale bar:50μm).

Supplementary Figure 4

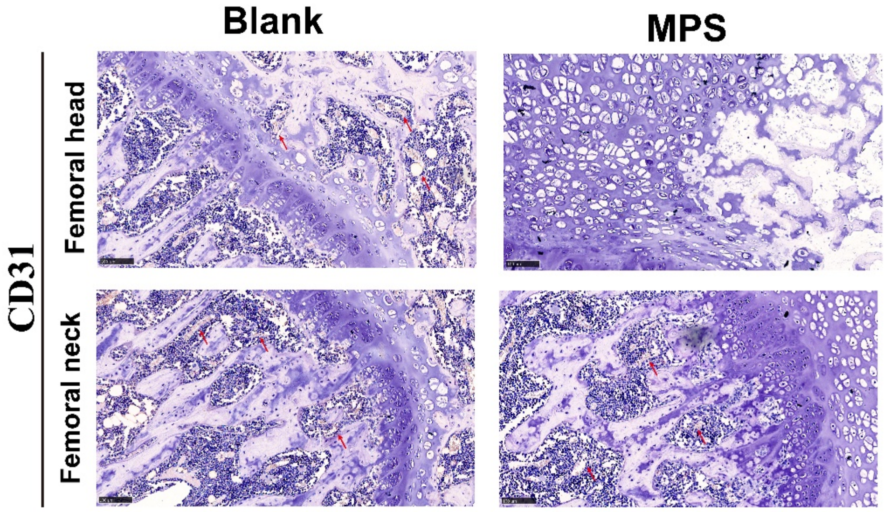

Supplementary Figure 4. Representative CD31 staining images of the femoral head and the femoral neck of rats in Blank and MPS groups (Scale bar:100μm)

Supplementary Figure 5：Original Western Blot images

Fig 1D

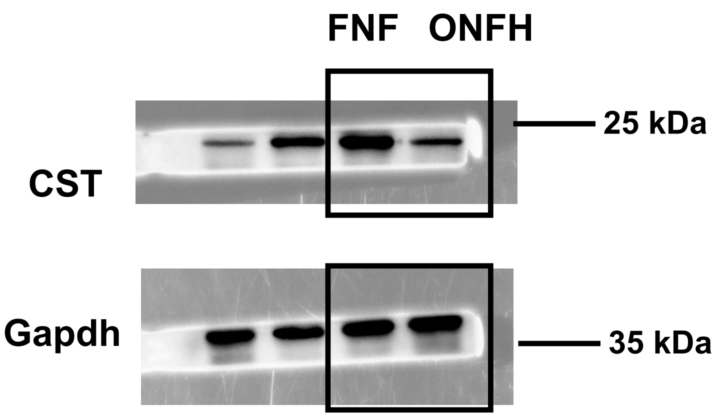

Fig 1H

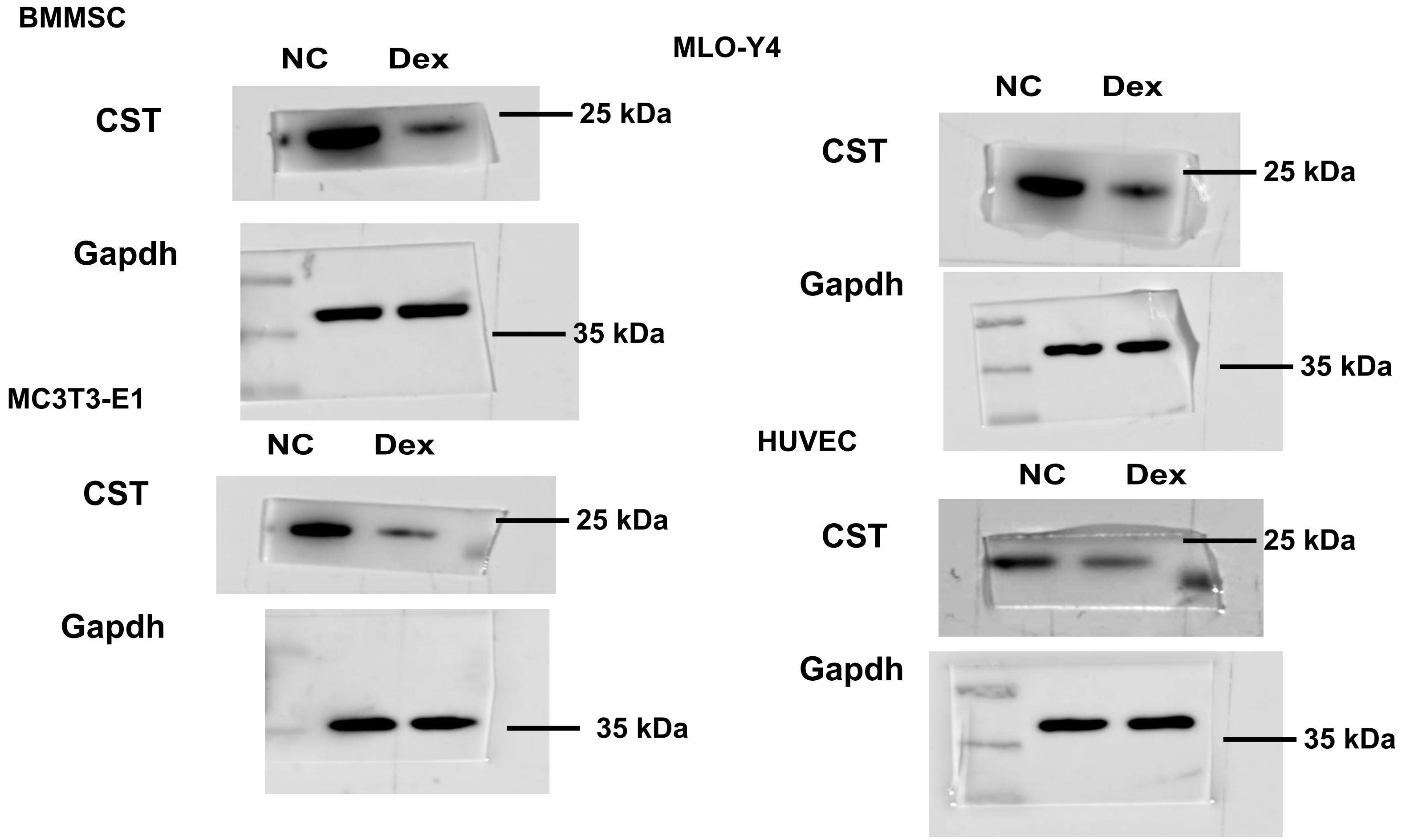

Fig 4A

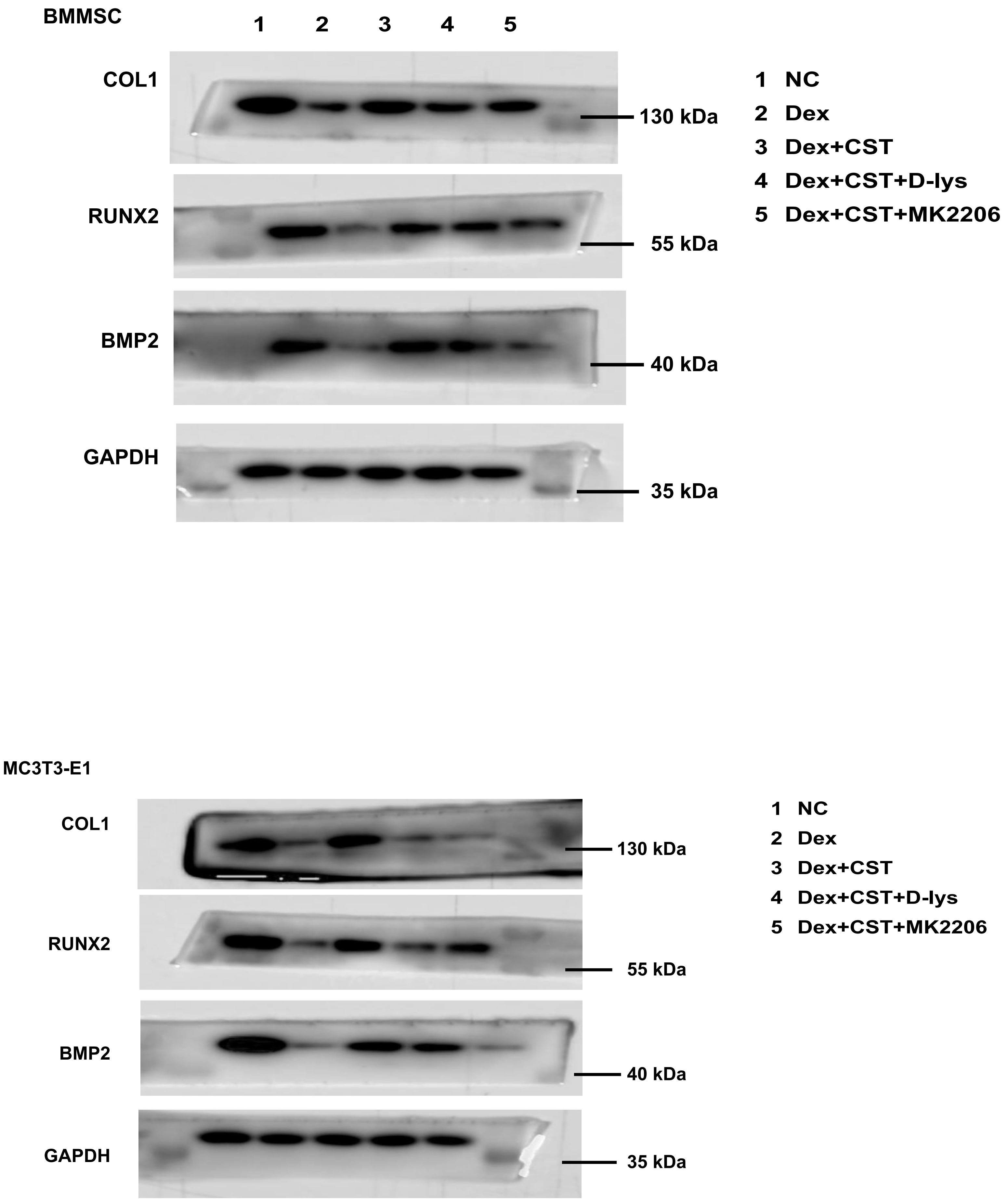
BMMSC
1 2 3 4 5
COL1
130 kDa
RUNX2
55 kDa
BMP2
40 kDa
GAPDH
35 kDa
1 NC
2 Dex
3 Dex+CST
4 Dex+CST+D-lys
5 Dex+CST+MK2206
MC3T3-E1
COL1
130 kDa
RUNX2
55 kDa
BMP2
40 kDa
GAPDH
35 kDa
1 NC
2 Dex
3 Dex+CST
4 Dex+CST+D-lys
5 Dex+CST+MK2206

Fig 5C

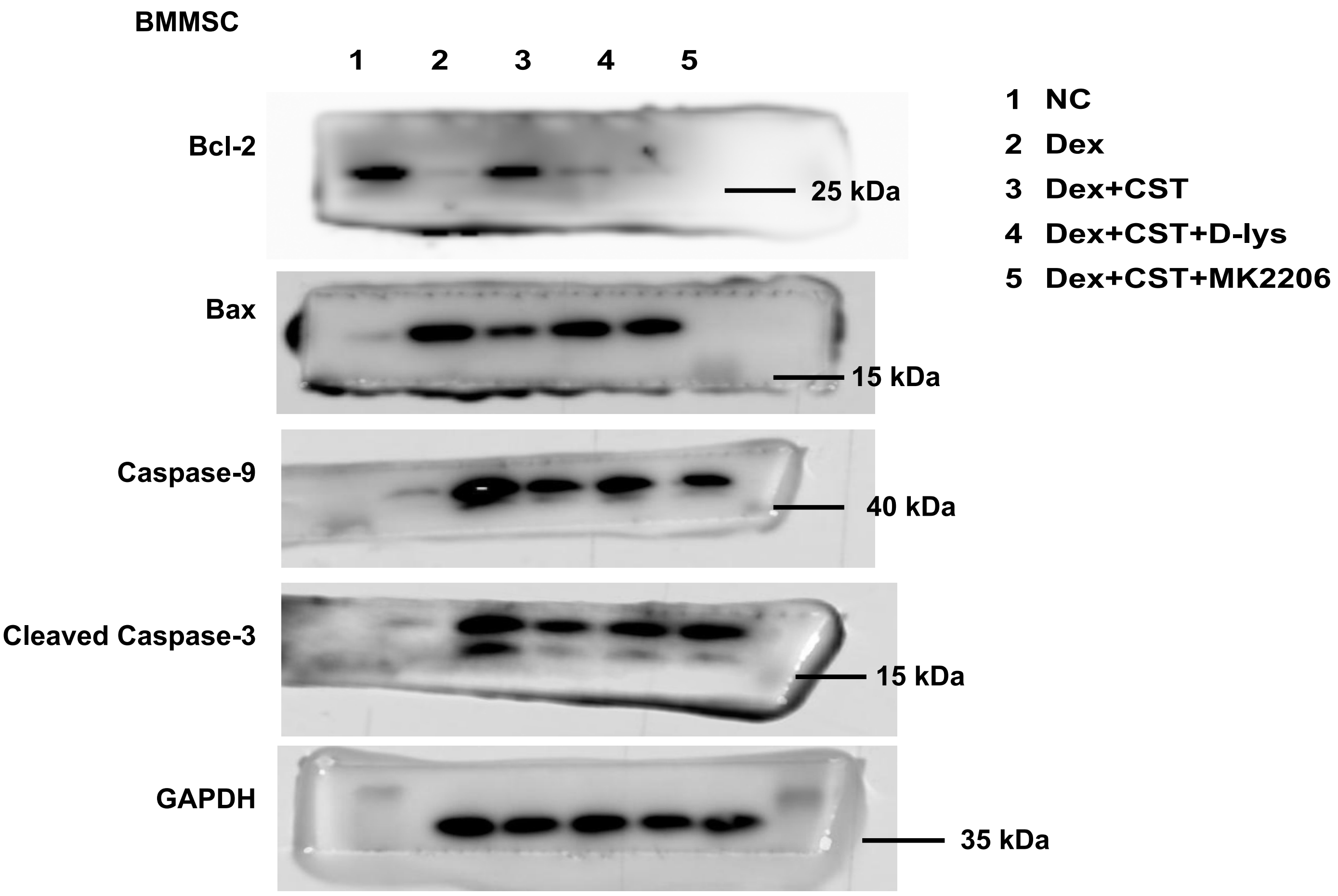
BMMSC
1 2 3 4 5
Bcl-2
25 kDa
Bax
15 kDa
Caspase-9
40 kDa
Cleaved Caspase-3
15 kDa
GAPDH
35 kDa
1 NC
2 Dex
3 Dex+CST
4 Dex+CST+D-lys
5 Dex+CST+MK2206

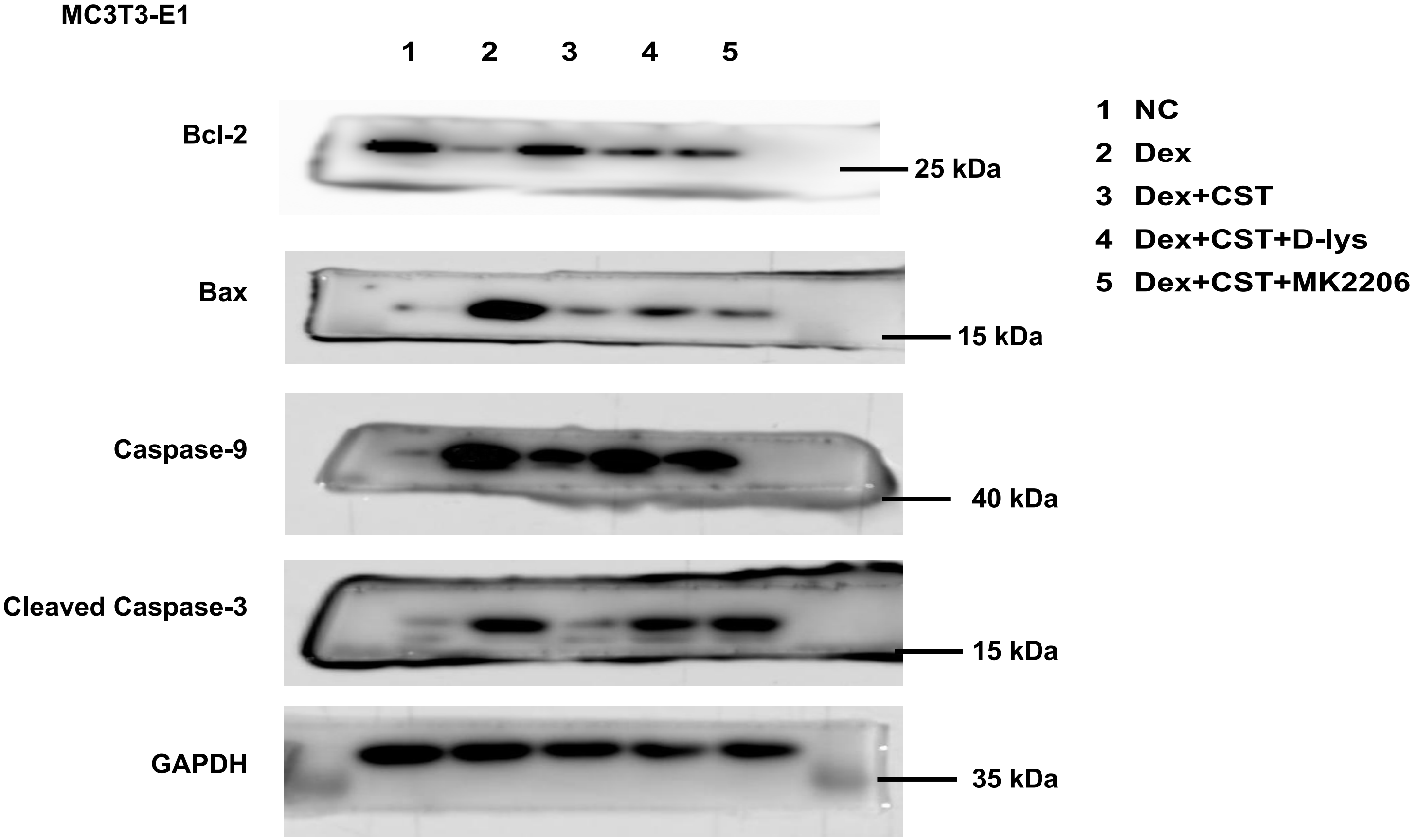
MC3T3-E1
1 2 3 4 5
Bcl-2
25 kDa
Bax
15 kDa
Caspase-9
40 kDa
Cleaved Caspase-3
15 kDa
GAPDH
35 kDa
1 NC
2 Dex
3 Dex+CST
4 Dex+CST+D-lys
5 Dex+CST+MK2206

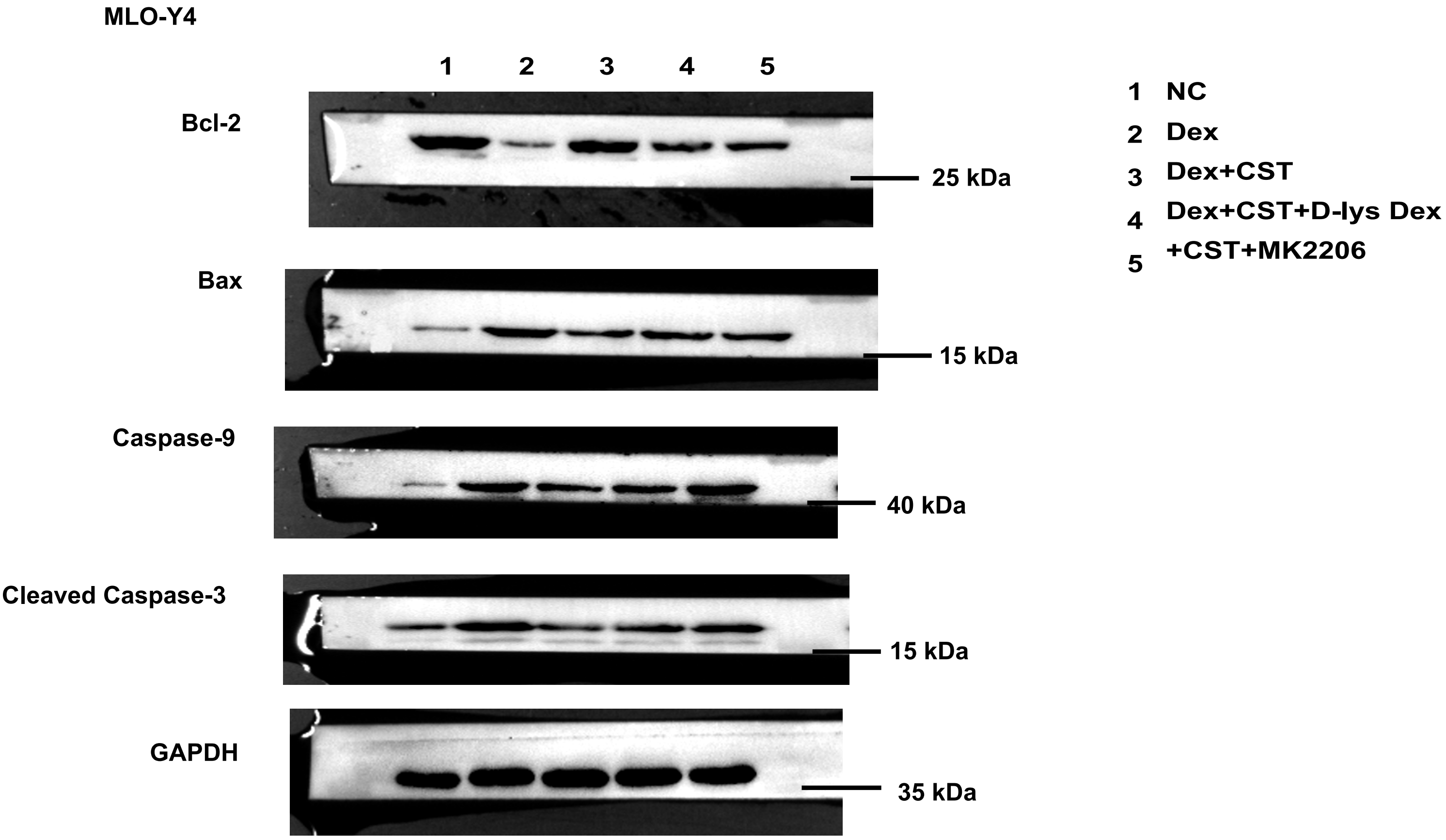
MLO-Y4
1
2
3
4
5
Bcl-2
25 kDa
Bax
15 kDa
Caspase-9
40 kDa
Cleaved Caspase-3
15 kDa
GAPDH
35 kDa
1 NC
2 Dex
3 Dex+CST
4 Dex+CST+D-lys Dex
5 +CST+MK2206

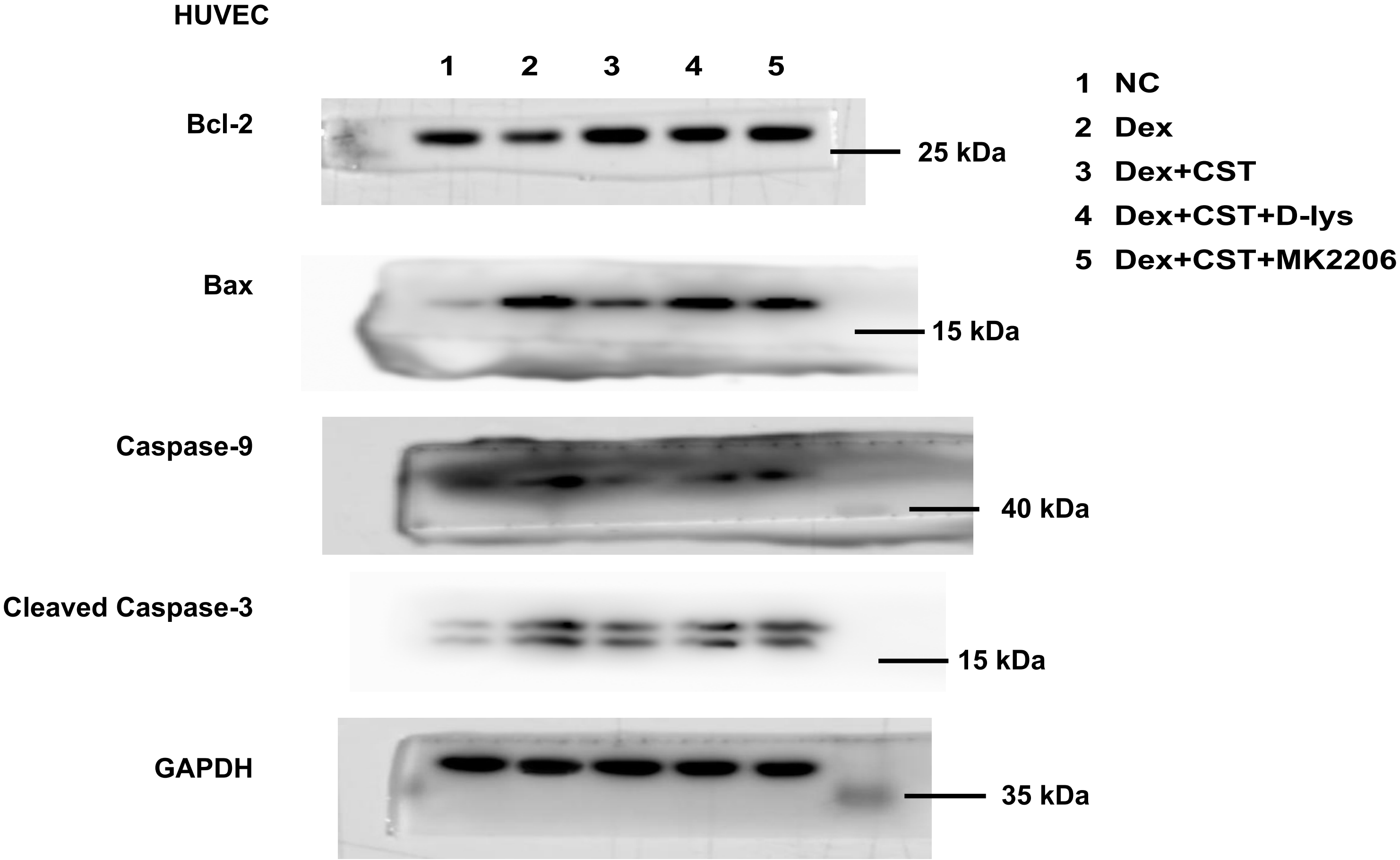
HUVEC
1
2
3
4
5
Bcl-2
25 kDa
Bax
15 kDa
Caspase-9
40 kDa
Cleaved Caspase-3
15 kDa
GAPDH
35 kDa
1 NC
2 Dex
3 Dex+CST
4 Dex+CST+D-lys
5 Dex+CST+MK2206

Fig 7B

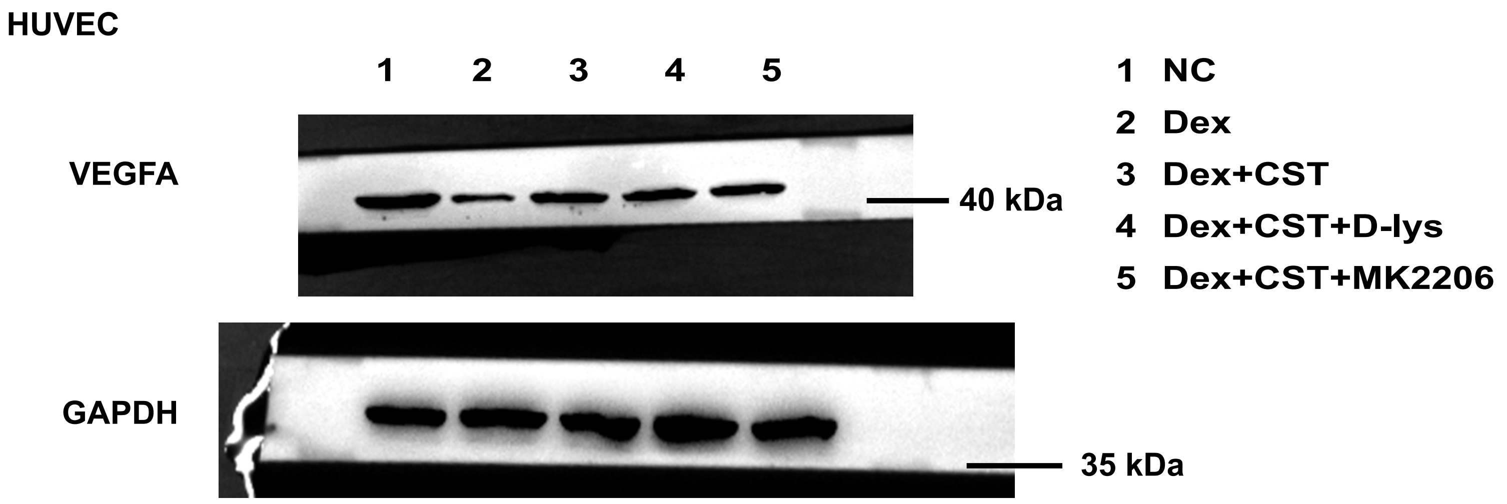
HUVEC
1
2
3
4
5
VEGFA
40 kDa
GAPDH
35 kDa
1 NC
2 Dex
3 Dex+CST
4 Dex+CST+D-lys
5 Dex+CST+MK2206

Supplementary Figure 6：The gating strategy

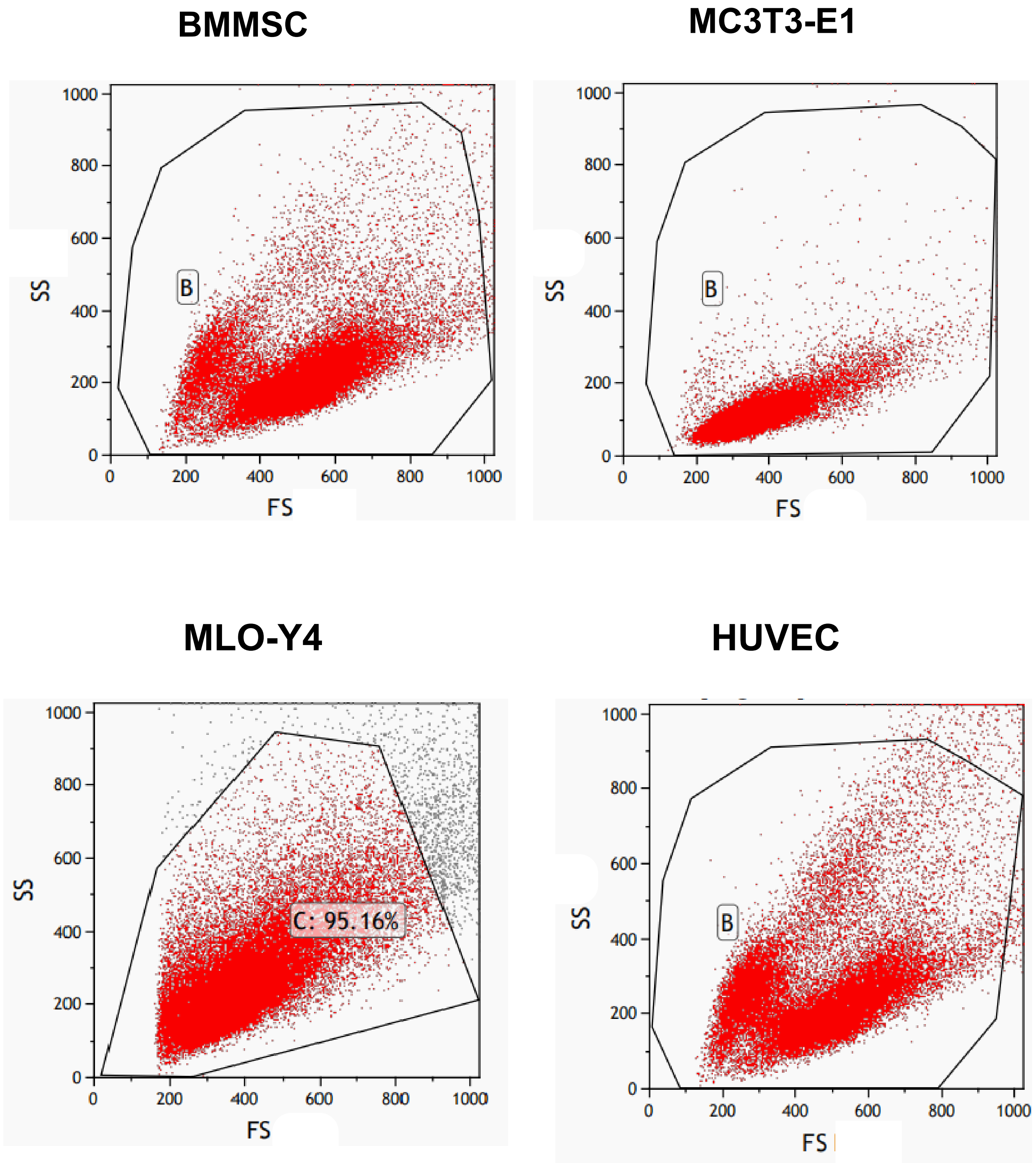

Supplement: Supplementary file 1 — Supplementary Information [file 42003_2024_5795_MOESM1_ESM.pdf]
