# Supplementary material for: Cortistatin prevents glucocorticoid-associated osteonecrosis of the femoral head via the GHSR1a/Akt pathway
Source: Commun Biol. 2024 Jan 26;7:132. doi: 10.1038/s42003-024-05795-5 (PMC10817896; doi:10.1038/s42003-024-05795-5)
Supplement: Supplementary file 4 — Reporting Summary [file 42003_2024_5795_MOESM4_ESM.pdf]

nature portfolio

Corresponding author(s): Hao Wang, Shuai Zhang, Yuhua Li, Weiwei Li

Last updated by author(s): Jan 3, 2024

# Reporting Summary

Nature Portfolio wishes to improve the reproducibility of the work that we publish. This form provides structure for consistency and transparency in reporting. For further information on Nature Portfolio policies, see our Editorial Policies and the Editorial Policy Checklist.

## Statistics

For all statistical analyses, confirm that the following items are present in the figure legend, table legend, main text, or Methods section.

| n/a | Confirmed | |
|---|---|---|
| ☐ | ☒ | The exact sample size (*n*) for each experimental group/condition, given as a discrete number and unit of measurement |
| ☐ | ☒ | A statement on whether measurements were taken from distinct samples or whether the same sample was measured repeatedly |
| ☐ | ☒ | The statistical test(s) used AND whether they are one- or two-sided<br>*Only common tests should be described solely by name; describe more complex techniques in the Methods section.* |
| ☒ | ☐ | A description of all covariates tested |
| ☒ | ☐ | A description of any assumptions or corrections, such as tests of normality and adjustment for multiple comparisons |
| ☐ | ☒ | A full description of the statistical parameters including central tendency (e.g. means) or other basic estimates (e.g. regression coefficient) AND variation (e.g. standard deviation) or associated estimates of uncertainty (e.g. confidence intervals) |
| ☐ | ☒ | For null hypothesis testing, the test statistic (e.g. *F*, *t*, *r*) with confidence intervals, effect sizes, degrees of freedom and *P* value noted<br>*Give P values as exact values whenever suitable.* |
| ☒ | ☐ | For Bayesian analysis, information on the choice of priors and Markov chain Monte Carlo settings |
| ☒ | ☐ | For hierarchical and complex designs, identification of the appropriate level for tests and full reporting of outcomes |
| ☒ | ☐ | Estimates of effect sizes (e.g. Cohen's *d*, Pearson's *r*), indicating how they were calculated |

*Our web collection on statistics for biologists contains articles on many of the points above.*

## Software and code

Policy information about availability of computer code

| | |
|---|---|
| Data collection | No software was used. |
| Data analysis | Data analysis was conducted using GraphPad Prism 7.0, Microsoft Excel |

For manuscripts utilizing custom algorithms or software that are central to the research but not yet described in published literature, software must be made available to editors and reviewers. We strongly encourage code deposition in a community repository (e.g. GitHub). See the Nature Portfolio guidelines for submitting code & software for further information.

## Data

Policy information about availability of data

All manuscripts must include a data availability statement. This statement should provide the following information, where applicable:

- Accession codes, unique identifiers, or web links for publicly available datasets
- A description of any restrictions on data availability
- For clinical datasets or third party data, please ensure that the statement adheres to our policy

The reagents used in the current study are available from the corresponding author on reasonable request.

## Human research participants

Policy information about studies involving human research participants and Sex and Gender in Research.

| | |
|---|---|
| Reporting on sex and gender | Human femoral head tissue and sera were obtained from total hip arthroplasties at Qilu Hospital Shandong University,and he sex and gender were not considered in this study design |
| Population characteristics | See above. |
| Recruitment | Human femoral heads and sera were obtained from total hip arthroplasties at Qilu Hospital Shandong University,and patients involved in the study provided consent. |
| Ethics oversight | The Medical Ethical Committee of Qilu Hospital of Shandong University |

Note that full information on the approval of the study protocol must also be provided in the manuscript.

# Field-specific reporting

Please select the one below that is the best fit for your research. If you are not sure, read the appropriate sections before making your selection.

☒ Life sciences ☐ Behavioural & social sciences ☐ Ecological, evolutionary & environmental sciences

For a reference copy of the document with all sections, see nature.com/documents/nr-reporting-summary-flat.pdf

# Life sciences study design

All studies must disclose on these points even when the disclosure is negative.

| | |
|---|---|
| Sample size | We determined the number of experimental animals according to the references DOI: 10.7150/thno.17450; DOI: 10.7150/ijbs.16150.Animal and cell n number were indicated in each figures. |
| Data exclusions | No data were excluded from the analysis. |
| Replication | All attempts at replication were successful. |
| Randomization | The rats were randomly divided into three groups. The sham group was used as a control, and GC-induced ONFH models were established in the other two groups: the PBS treatment control group and the CST treatment group. |
| Blinding | The investigators were blinded to group allocation during data collection and analysis. |

# Reporting for specific materials, systems and methods

We require information from authors about some types of materials, experimental systems and methods used in many studies. Here, indicate whether each material, system or method listed is relevant to your study. If you are not sure if a list item applies to your research, read the appropriate section before selecting a response.

### Materials & experimental systems

| n/a | Involved in the study | |
|---|---|---|
| ☐ | ☒ | Antibodies |
| ☐ | ☒ | Eukaryotic cell lines |
| ☒ | ☐ | Palaeontology and archaeology |
| ☐ | ☒ | Animals and other organisms |
| ☒ | ☐ | Clinical data |
| ☒ | ☐ | Dual use research of concern |

### Methods

| n/a | Involved in the study | |
|---|---|---|
| ☒ | ☐ | ChIP-seq |
| ☐ | ☒ | Flow cytometry |
| ☒ | ☐ | MRI-based neuroimaging |

## Antibodies

| | |
|---|---|
| Antibodies used | The primary antibodies, including CST(ab48236,Biotin Rabbit polyclonal to Cortistatin, Abcam Cambridge, UK), osteocalcin(ab93876, Rabbit polyclonal to Osteocalcin, Abcam Cambridge, UK), VEGF (ab1316, Mouse monoclonal [VG-1] to VEGFA, Abcam Cambridge, UK)and CD31 (ab28364, Rabbit polyclonal to CD31, Abcam Cambridge, UK)were used for immunohistochemical staining.The anti-VEGFA (Product Number: 50661; Cell Signaling Technology, USA), anti-caspase-9 (Product Number: 9508; Cell Signaling Technology, |

USA), anti-cleaved-caspase-3 (Product Number: 9661; Cell Signaling Technology, USA)), anti-Bcl-2 (Product Number: 15071; Cell Signaling Technology, USA), anti-Bax (Product Number: 14796; Cell Signaling Technology, USA), anti- COL1A1 (Product Number: 72026; Cell Signaling Technology, USA), anti-BMP2 (Cat No : 66383-1-Ig,Proteintech, P.R.C), anti-Runx2(Product Number: 12556; Cell Signaling Technology, USA), and anti-GAPDH(Product Number: 5174; Cell Signaling Technology, USA) were used for WB.

| | |
|---|---|
| Validation | The validation of commercial antibodies used in this study were posted in manufacturer s website. |

## Eukaryotic cell lines

Policy information about cell lines and Sex and Gender in Research

| | |
|---|---|
| Cell line source(s) | Murine bone marrow mesenchymal stem cells (BMMSCs), mouse embryo osteoblast precursor cells (MC3T3-E1Cs) and human microvascular endothelial cells (HUVECs) were purchased from Zhong Qiao Xin Zhou Biotechnology Co., Ltd. (Shanghai, China). |
| Authentication | The STR identification certificates were attached to the cell purchase |
| Mycoplasma contamination | The detection of mycoplasma in all cell lines was negative |
| Commonly misidentified lines (See ICLAC register) | Not applicable |

## Animals and other research organisms

Policy information about studies involving animals; ARRIVE guidelines recommended for reporting animal research, and Sex and Gender in Research

| | |
|---|---|
| Laboratory animals | Sprague–Dawley (SD) rats (10 weeks old, 300±30g) |
| Wild animals | The study did not involve wild animals. |
| Reporting on sex | The sex was not considered in study design. |
| Field-collected samples | The study did not involve samples collected from the field. |
| Ethics oversight | All experimental procedures were completed under the Care and Use of Laboratory Animals guidance and approved by the Animal Ethics Committee of Qilu Hospital Shandong University. |

Note that full information on the approval of the study protocol must also be provided in the manuscript.

## Flow Cytometry

### Plots

Confirm that:

☒ The axis labels state the marker and fluorochrome used (e.g. CD4-FITC).

☒ The axis scales are clearly visible. Include numbers along axes only for bottom left plot of group (a 'group' is an analysis of identical markers).

☒ All plots are contour plots with outliers or pseudocolor plots.

☐ A numerical value for number of cells or percentage (with statistics) is provided.

### Methodology

| | |
|---|---|
| Sample preparation | Cultured BMMSCs, MC3T3-E1Cs and MLO-Y4Cs (purchased from Zhong Qiao Xin Zhou Biotechnology Co., Ltd. Shanghai, China) from each indicated group were detected by flow cytometry. Cells were stained with propidium iodide (PI) and Annexin V-FITC for 15 min at room temperature in the dark in accordance with the protocol of the BD Pharmingen FITC Annexin V Apoptosis Detection Kit I (BD Biosciences, USA) |
| Instrument | CytoFLEX S flow cytometer (Beckman Coulter, USA). |
| Software | FlowJo software. |
| Cell population abundance | BMMSCs, MC3T3-E1Cs and MLO-Y4Cs were used. |
| Gating strategy | The untreated cells were set on the FSC/SSC dot pattern of unstained control cells . And the singlet population were further confirmed by FSC-H/FSC-A dot pattern of unstained control cells. Three cell subpopulations identified from the singlet population of Annexin V-FITC and PI staining A/R cells with unstained control cells as reference. |

☒ Tick this box to confirm that a figure exemplifying the gating strategy is provided in the Supplementary Information.
